# Comparative analysis of protein-protein interaction networks in metastatic breast cancer

**DOI:** 10.1371/journal.pone.0260584

**Published:** 2022-01-19

**Authors:** Hossein Hozhabri, Roxana Sadat Ghasemi Dehkohneh, Seyed Morteza Razavi, S. Mostafa Razavi, Fatemeh Salarian, Azade Rasouli, Jalil Azami, Melika Ghasemi Shiran, Zahra Kardan, Negar Farrokhzad, Arsham Mikaeili Namini, Ali Salari

**Affiliations:** 1 Institute of Biochemistry and Biophysics, University of Tehran, Tehran, Iran; 2 Salari Institute of Cognitive and Behavioral Disorders (SICBD), Karaj, Alborz, Iran; 3 Systems Biology Research Lab, Bioinformatics Group, Systems Biology of the Next Generation Company (SBNGC), Qom, Iran; 4 Department of Cell and Molecular Biology and Microbiology, Faculty of Biological Science and Technology, University of Isfahan, Isfahan, Iran; 5 Department of Cell and Molecular Biology, Faculty of Biological Sciences, Kharazmi University, Tehran, Iran; 6 Department of Chemical, Biomolecular and Corrosion Engineering, The University of Akron, Akron, Ohio, United States of America; 7 Department of Cellular Biotechnology, Cell Science Research Center, Royan Institute for Biotechnology, ACECR, Isfahan, Iran; 8 Department of Toxicology and Pharmacology, Faculty of Pharmacy, Tehran University of Medical Sciences, Tehran, Iran; 9 Faculty of Veterinary Medicine, Urmia University, Urmia, Iran; 10 Department of Biology, Faculty of Sciences, Central Tehran Branch, Islamic Azad University, Tehran, Iran; 11 Department of Cellular Molecular Biology, Faculty of Life Science and Biotechnology, Shahid Beheshti University, Tehran, Iran; 12 Department of Biology, Faculty of Sciences, University of Guilan, Rasht, Iran; 13 Department of Animal Biology, Faculty of Biological Sciences, Kharazmi University, Tehran, Iran; 14 Department of Stem Cells and Developmental Biology, Cell Science Research Center, Royan Institute for Stem Cell Biology and Technology, ACECR, Tehran, Iran; Northumbria University, UNITED KINGDOM

## Abstract

Metastatic lesions leading causes of the majority of deaths in patients with the breast cancer. The present study aimed to provide a comprehensive analysis of the differentially expressed genes (DEGs) in the brain (MDA-MB-231 BrM2) and lung (MDA-MB-231 LM2) metastatic cell lines obtained from breast cancer patients compared with those who have primary breast cancer. We identified 981 and 662 DEGs for brain and lung metastasis, respectively. Protein-protein interaction (PPI) analysis revealed seven shared (*PLCB1*, *FPR1*, *FPR2*, *CX3CL1*, *GABBR2*, *GPR37*, and *CXCR4)* hub genes between brain and lung metastasis in breast cancer. Moreover, *GNG2* and *CXCL8*, *C3*, *and PTPN6* in the brain and *SAA1* and *CCR5* in lung metastasis were found as unique hub genes. Besides, five co-regulation of clusters via seven important co-expression genes (COL1A2, LUM, SPARC, THBS2, IL1B, CXCL8, THY1) were identified in the brain PPI network. Clusters screening followed by biological process (BP) function and pathway enrichment analysis for both metastatic cell lines showed that complement receptor signalling, acetylcholine receptor signalling, and gastric acid secretion pathways were common between these metastases, whereas other pathways were site-specific. According to our findings, there are a set of genes and functional pathways that mark and mediate breast cancer metastasis to the brain and lungs, which may enable us understand the molecular basis of breast cancer development in a deeper levele to the brain and lungs, which may help us gain a more complete understanding of the molecular underpinnings of breast cancer development.

## Introduction

Breast cancer is the most frequent malignancy among females, followed by colorectal and lung cancer in terms of incidence and vice versa in terms of mortality. Overall, breast cancer is the second most common cancer following lung cancer [1]. Triple-negative breast cancer (TNBC) is a subtype of breast cancer characterized by lack of an estrogen receptor (ER), progesterone receptor (PR), and *HER2* expression, with a high frequency of visceral metastases and worse prognosis [2]. Clinically, metastasis is the major cause of death from cancer. Dissemination of tumour cells to certain organs is an intentional process known as “metastatic organotropism”. This process is controlled by various factors such as the cancer subtype, molecular features of cancer cells, host immune microenvironment, and cross-talk with local cells [3]. For instance, the host microenvironment is modified to form a pre-metastatic niche, which provides a protective environment for tumour growth in the host tissue before tumour dissemination [4].

Tumour metastasis is the main challenge in solid tumour oncology [5]. The most frequent sites of breast cancer metastasis include the bones (30%–60%) and lungs (21%–32%), while the liver (15%–32%) and brain (4%–10%) are less frequent [6]. Tumour genomic alterations such as gene expression levels and mutations play an important role in stimulating breast cancer metastasis. For instance, mutations in the TP53 tumour suppressor gene, which account for more than 50% of metastatic TNBC tumours, as well as PIK3CA, RB1, and PTEN genes, are more prevalent [7]. Liu et al. have reported that pathway enrichment levels of TP53-mutant breast cancer samples are significantly different from TP53-wildtype breast cancer specimens. They showed that immunogenic activity-associated pathways are altered in TP53-mutant BCs. For example, the SPNS2 (sphingolipid transporter 2) gene, which promotes tumour metastasis by regulating lymphocyte trafficking, has higher expression levels in TP53-mutant BCs compared with TP53-wildtype samples [8]. Moreover, the homologous recombination system, which is essential for maintaining DNA integrity, showed a deficiency in 10–20% of TNBC patients due to germline mutations of BRCA1/2 [9]. Another study demonstrated that DEGs and PPI networks are affected by the BRCA1/2 gene mutation in breast cancer samples. This study also reported that distant metastasis, aggressiveness, and protein interactions in many biological pathways are changed in BRCA1/2-mutated condition when compared with BRCA1/2-wild-type breast cancer samples [10]. In addition to genomic alterations, tumour microsatellite instability (MSI), which disrupts the DNA mismatch repair system, is another stimulant of breast cancer metastasis [7].

Despite breakthroughs in early diagnosis and better treatment of breast cancer that lead to improved survival, the median survival of breast cancer patients is approximately 4–5 months after the development of brain metastasis and 22 months after the onset of lung metastasis [11, 12]. Hence, the development of management strategies for early diagnosis and treatment of brain and lung metastases induced by breast cancer is of critical importance and would lead to improved quality of life [13].

An aberrant gene expression plays a pivotal role in tumorigenesis, progression, and metastasis of breast cancer [14]. For instance, Massaque and colleagues identified 18 genes, including *IL13Ra2*, *SPARC*, *MMP1*, and *MMP2*, which can lead to breast cancer metastasis to the lungs [15]. Lee et al. identified a panel of 22 genes such as *VCAM1*, *CXCL12*, and *MMP2* resulting in significant differential expressions between primary breast cancer and brain metastasis [16]. Furthermore, Engin et al. have detected seven hub nodes for both brain and lung metastases induced by breast cancer. Breast cancer was also linked to a higher incidence of infectious diseases and immune system activity in lung metastasis, while catabolism and transport were more prevalent in brain metastasis, according to this study [17].

The above-mentioned microarray data were utilized to identify DEGs and functional pathways associated with breast cancer-induced brain and lung metastases. Nevertheless, recently with the advancement of next-generation sequencing technologies, cancer early detection has become easier and faster [18]. The RNA-sequencing approach has several advantages over microarray data processing, including the ability to identify a greater proportion of genes encoding differentially expressed protein-coding genes, increased specificity and sensitivity, and a wider dynamic range [19, 20]. As a result of this cutting-edge analytical technique, a more solid option for fully investigating novel genes in the genetics area is accessible now.

RNA-sequencing data analysis on the human breast cancer cell line MDA-MB-231 has shown that *CXCR4*, *PLLP*, *VCAM1*, *SLC7A11*, *SLC8A2*, and *TNFSF4* genes are highly over-expressed in brain metastasis [21]. CHIP-sequencing and RNA-sequencing data analysis performed by Li et al. revealed that many biological pathways promote lung metastasis of breast cancer. These include cell migration, immune response, and other potential factors and regulators of metastasis such as LMO4 [22].

In the present study, we used RNA-sequencing gene expression data analysis to identify the potential genes and key pathways in brain and lung metastases from breast cancer. Our study provides a new deeper insight into betterunderstanding of these hub genes and the pathways involved in tumour progression. The obtained data have depicted that certain hub genes are correlated with brain and lung metastases from breast cancer, suggesting that they can potentially be used in novel therapeutic strategies.

## Materials and methods

### Data source

The MDA-MB-231 (ATCCHTB-26) cell line derived from human tissues and its metastatic subpopulations, BrM2 and LM2, were also appeared in Massaque analysis [23]. In this study, we elucidated the main differences between the primary and secondary breast cancer cell lines by comparing the transcriptional content of breast epithelial tissues in primary breast cancer with their metastases to the brain and lungs. Three replicates of each sample were used for the analysis. The RNA-seq FASTQ [24] files were downloaded from the Gene Expression Omnibus (GEO) [25] database (accession number: GSE138122).

### Data processing and analysis

Paired-end reads were aligned to the hg38 reference genome by using STAR aligner version 2.7.0 [26] with the default parameters and quant mode for gene counts. DEGs were obtained using DESeq2 [27]. The details of RNA-sequencing data processing methods were performed according to a study conducted by Cail et al. [28]. An adjusted P-value of ≤0.05 was considered significant. To extract the DEGs, log2 fold changes greater than +2 and less than -2 were set as the cutoff values. Volcano plots were used to visualize the concentration of significant DEGs and site specific comparison of genes expression. Visualization of overlapping DEGs was illustrated using Venn diagrams of the lung and brain metastatic samples compared with non-metastatic breast cancer samples by using the VennDiagram package in R [29].

#### Gene ontology (GO) and Kyoto Encyclopedia of Genes and Genomes (KEGG) pathway enrichment analysis

Gene Ontology (GO) is categorized into three main parts; biological process (BP), molecular function (MF), and cellular component (CC). BP and Kyoto Encyclopedia of Genes and Genomes (KEGG) [30] pathway analyses for the DEGs from the clusters were performed using an online functional annotation tool named Enrichr [31] (https://amp.pharm.mssm.edu/Enrichr) to provide a comprehensive understanding of the biological information of the genes, proteins, and their associated pathways. An adjusted P-value of <0.05 was set as the cutoff criteria for screening the BP and KEGG enrichment pathways. The BP and KEGG results were ranked based on the Rich factor, that is the ratio of the DEG numbers and the numbers of genes that were annotated in the associated pathway. Accordingly, the degree of enrichment increases with an increase in the Rich factor.

#### Protein-protein interaction (PPI) network construction and subnetwork analysis

To predict the interaction pattern of DEGs in brain and lung metastases, the PPI of DEGs was visualized using the Search Tool for the Retrieval of Interacting Genes/Proteins (STRING) database (https://string-db.org) with a combined score of >0.7. STRING currently covers 24.6 million proteins from 5090 organisms [32]. In general, there are three types of confidence scores for PPIs: 1) low confidence: score < 0.4, 2) medium confidence: 0.4< score <0.7, and 3) high confidence: score >0.7 [33]. In the present study, we selected a high confidence score to eliminate PPIs with low probability/significance and obtain more reliable results. However, we also utilized the BioPlex database (https://bioplex.hms.harvard.edu) that includes nearly 120,000 interactions of nearly 15,000 proteins making it the most comprehensive experimentally derived model of the human interactome [34]. Protein-protein interaction (PPI) networks constructed with a confidence score of ≥0.7 from the STRING and BioPlex database were then merged and visualized by Cytoscape software (version 3.7.2) [35]. We removed the protein nodes with no interactions with other proteins. In the PPI network, the genes served as the nodes and the edges represented the associated interactions. The connectivity degree of each node, which indicates the number of interactions of the corresponding gene, was examined by the CentiScaPe plugin [36] in Cytoscape. Nodes with a degree of connectivity ≥15 were labelled as hub genes. Moreover, protein complex analysis was carried out by the Molecular Complex Detection plugin (MCODE) [37], to identify significant primary clusters of this large PPI network. The advanced options were set as follows: degree cutoff = 2, node score cutoff = 0.2, and K-Core = 2. A complex of protein with a score ≥5 was selected as significant sub-networks. Moreover, to identify which clusters may be co-regulated to other clusters, we performed co-expression analysis via STRING database to all genes of clusters. Then, the results of co-expression analysis were merged to clusters to find out which clusters have connections with other clusters via co-expression genes. This could be helpful in understanding and predicting the clusters that are co-regulated via co-expression genes in network analysis. Subsequently, the BP and KEGG pathway enrichment analyses of the DEGs in all clusters were performed using the Enrichr tool [31].

### Cancer dependency map (DepMap)

Since all the analysis of the current study was performed on MDA-MB-231 cell lines, it was necessary to evaluate the dependency score of selected hub genes for the MDA-MB-231 cell line. Genetic dependencies of hub genes for MDA-MB-231 were surveyed using DepMap, an online platform accessible at https://depmap.org/portal/download. DepMap is a cancer dependency map that identifies the effect of gene knockdown viability for cancer cell lines [38]. Two downloaded databases from DepMap were used in this study: the combined RNAi (D2_combined_gene_dep_scores.csv) [39] and the CRISPR (Avana) Public 20Q2 (Achilles_gene_effect.csv) [40]. In both databases, a lower score represents a higher dependency of hub genes for the viability of the MDA-MB-231 cell line and zero indicates no dependency [38].

### Survival analysis of hub genes

Clinical outcome analysis of the hub genes in breast cancer was carried out by the Kaplan-Meier plotter database (http://kmplot.com/analysis/), which enabled us to assess the effect of 54 000 genes on survival in 21 cancer types. These cover 6234 breast, 2190 ovarian, 3452 lung, and 1440 gastric cancer patients’ overall survival information. In this database, the gene expression data and overall survival information are based on GEO, EGA, and TCGA. The survival analysis was carried out for all breast cancer subtypes, not just TNBC. Patients were categorized into two groups based on the median of each hub gene expression in the Kaplan-Meier plotter in order to perform overall survival analysis. The results from this tool can assist us in cancer biomarker assessment in order to improve clinical decisions and health care policies.

## Results

### Differentially expressed gene (DEG) analysis in brain and lungs metastatic breast cancer cells

**Fig 1:**
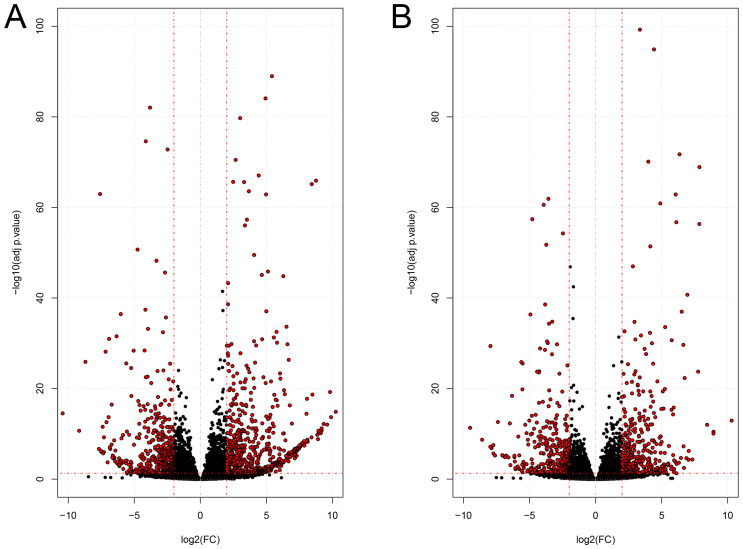
Dispersion patterns of differential expression genes (DEGs): **(A)** Volcano plot of DEGs in brain metastasis from breast cancer. **(B)** Volcano plot of DEGs in lung metastasis from breast cancer. The x-axis represents the log2 fold change and the y-axis represents the negative base 10 logarithm base 10 of the adjusted P-value. The cutoff criteria were the adjusted P-value ≤0.05 and log2 fold change higher than +2 and less than -2. The red dashed lines indicate log2 fold change ≤ -2 and ≥ +2 for the down-regulated and up-regulated genes, respectively.

**Fig 2:**
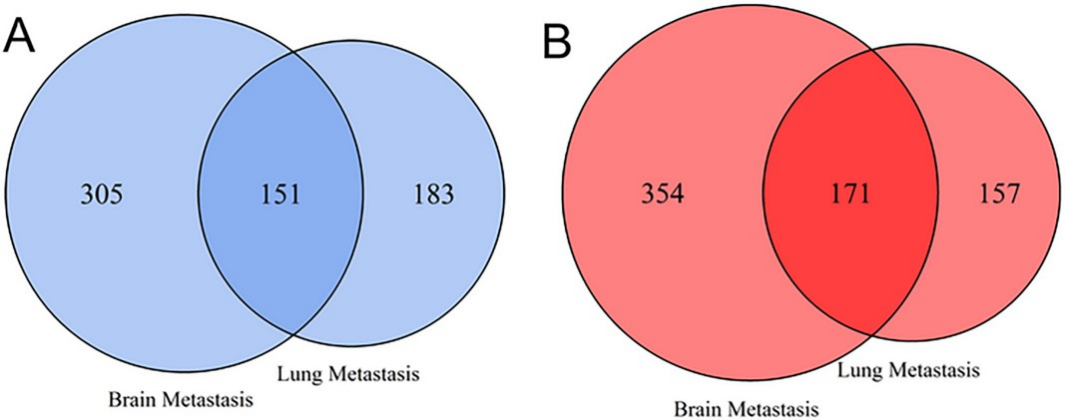
Venn diagram that shows the overlap of differentially expressed genes (DEGs) between the brain and lung metastases from breast cancer: **(A)** A total of 456 and 334 down-regulated DEGs were identified for brain and lung metastases, respectively, among which there were 151 common DEGs. **(B)** A total of 525 and 328 up-regulated DEGs were explored for brain and lung metastases, respectively, among which 171 DEGs were shared.

The gene expression profile of brain metastatic breast cancer (BrM2) and lung metastatic breast cancer (LM2) cell lines of human breast cancer patients were obtained from RNA sequencing analysis (S1 Table). As shown in Fig 1 and S2 Table, we obtained 981 DEGs (525 up-regulated and 456 down-regulated) for primary breast cancer to the BrM2 cell line (S3 Table) and 662 DEGs (328 up-regulated and 334 down-regulated) for primary breast cancer to the LM2 cell lines using log2 fold change ≤ -2 and ≥ +2 and adjusted p-value ≤ 0.05 (S4 Table). The Venn diagrams of these DEGs depicted in Fig 2 indicate that 171 up-regulated genes (S5A Table) and 151 down-regulated genes (S5B Table) were shared between the two metastatic cell lines, i.e. BrM2 and LM2.

### Protein-protein interaction (PPI) network construction

**Fig 3:**
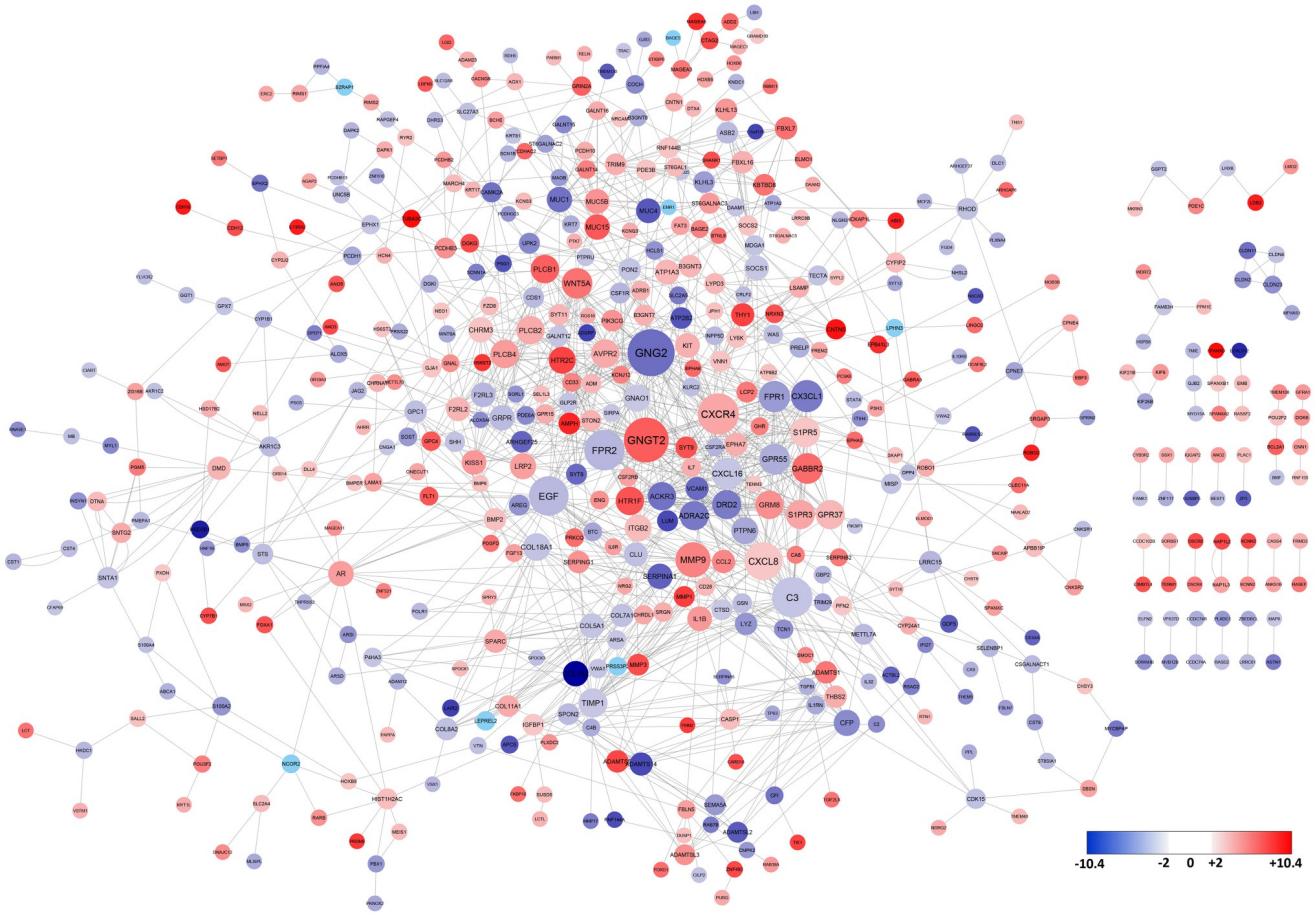
The Constructed PPI network with the DEGs for brain metastasis from breast cancer. Disconnected nodes are hidden in the network. The size of each node represents the degree of connectivity for identifying the key hub genes. The red nodes are up-regulated genes, while the blue nodes represent the down-regulated genes.

**Fig 4:**
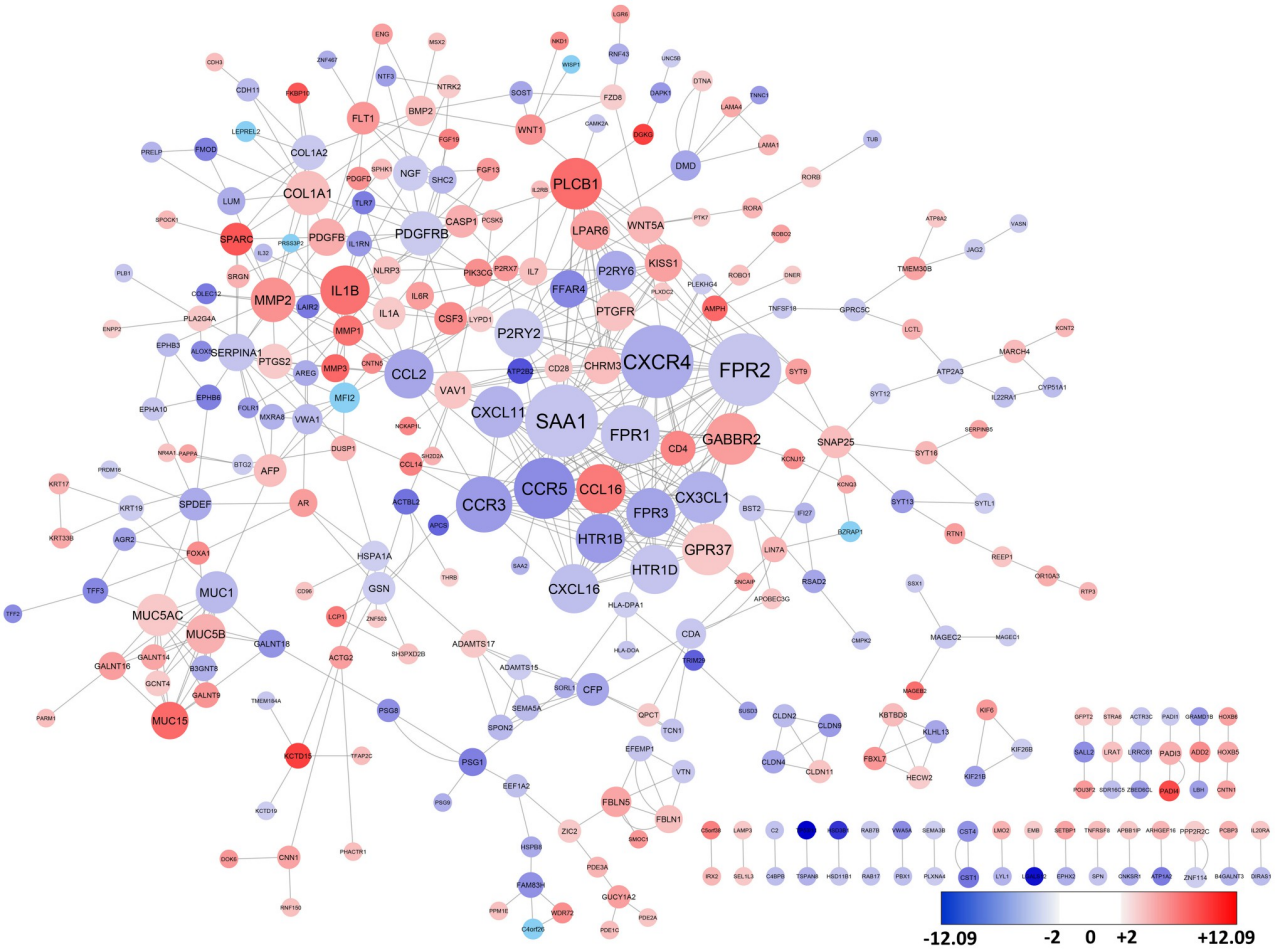
The Constructed PPI network with the DEGs for lung metastasis from breast cancer. Disconnected nodes were hidden in the network. Key hub nodes were highlighted with a larger size based on the degree of connectivity. Red and blue circles indicate up-regulated and down-regulated genes, respectively.

We used the STRING and BioPlex proteins databases to construct a PPI network. To calculate the interaction confidence in the network, each edge is assigned a score as the edge weight. This score represents the estimated probability that a given interaction is biologically meaningful. PPIs with a confidence score higher than 0.7 were selected to ensure the quality of interactions and minimize false-positive results. The PPI data for DEGs in the breast cancer metastases from the BrM2 cell line revealed 429 nodes and 1072 edges in the STRING database and 230 nodes and 247 edges in the BioPlex database. Merging these two networks resulted in a new network with 529 nodes and 1319 edges (Fig 3). For the brain metastasis cell line, 34 genes are reported as the hub genes by screening the nodes with a degree of connectivity higher than 15 (Table 1A). The degree of connectivity for G protein subunit gamma 2 (*GNG2*) and G protein subunit gamma transduction 2 (*GNGT2*) were > 40 in the brain metastases. Also, 27 nodes were reported as unique for the BrM2 cell line that was not found among DEGs of the lung metastasis LM2 cell line. We identified 240 nodes and 500 edges from the STRING database with a confident score ≥0.7 and 108 nodes and 81 edges from the BioPlex database, which were merged to form 300 nodes and 581 edges for the LM2 cell line (Fig 4). Analysis of the protein connectivity showed that the formyl peptide receptor 2 (FPR2), CXC motif chemokine receptor 4 (CXCR4), serum amyloid A 1 (SAA1), C-C motif chemokine receptor 5 (CCR5), formyl peptide receptor 1 (FPR1), C-C motif chemokine receptor 3 (CCR3), C-X3-C Motif Chemokine Ligand 1 (CX3CL1), C-X-C Motif Chemokine Ligand 11(CXCL11), G Protein-Coupled Receptor 37 (GPR37), Gamma-Aminobutyric Acid Type B Receptor Subunit 2 (GABBR2) and phospholipase C beta 1 (PLCB1) were hub nodes with degrees of ≥15 in breast cancer LM2 cell line (Table 1B). Notably, four hub genes (SAA1, CCR3, CCR5 and CXCL11) were unique among the breast cancer LM2 cell line; they were not identified among DEGs from the metastatic BrM2 cell line. There were seven shared hub nodes (FPR2, CXCR4, FPR1, CX3CL1, GPR37, GABBR2 and PLCB1) between metastatic breast cancer cell lines.

**Table 1:** Nodes with the highest degree of connectivity in the PPI network (degree ≥15). **(A)** Hub nodes extracted from the brain metastatic breast cancer PPI network. **(B)** Hub nodes obtained from the lung metastatic breast cancer PPI network.

A. Hub genes in the brain metastatic breast cancer			B. Hub genes in the lung metastatic breast cancer		
Gene symbol	Degree	Expression alteration	Gene symbol	Degree	Expression alteration
GNG2	43	Down-regulated	FPR2*	24	Down-regulated
GNGT2	40	Up-regulated	CXCR4*	24	Down-regulated
CXCR4*	35	Up-regulated	SAA1	24	Down-regulated
FPR2*	34	Down-regulated	CCR5	19	Down-regulated
C3	34	Down-regulated	FPR1*	18	Down-regulated
EGF	33	Down-regulated	CCR3	17	Down-regulated
CXCL8	31	Up-regulated	CX3CL1*	15	Down-regulated
MMP9	27	Up-regulated	CXCL11	15	Down-regulated
CX3CL1*	23	Down-regulated	GPR37*	15	Up-regulated
FPR1*	22	Down-regulated	GABBR2*	15	Up-regulated
CXCL16	22	Down-regulated	PLCB1*	15	Up-regulated
GPR55	21	Down-regulated			
WNT5A	21	Up-regulated			
GABBR2*	21	Up-regulated			
S1PR5	20	Up-regulated			
ADRA2C	19	Down-regulated			
DRD2	19	Down-regulated			
TIMP1	19	Down-regulated			
GPR37*	19	Up-regulated			
S1PR3	19	Up-regulated			
ACKR3	18	Down-regulated			
PLCB2	18	Up-regulated			
AVPR2	18	Up-regulated			
PLCB4	18	Up-regulated			
GRM8	18	Up-regulated			
PLCB1*	18	Up-regulated			
HTR1F	18	Up-regulated			
COL18A1	17	Down-regulated			
PTPN6	16	Down-regulated			
GNAO1	16	Down-regulated			
ITGB2	16	Up-regulated			
HTR2C	16	Up-regulated			
CFP	15	Down-regulated			
LRP2	15	Up-regulated			

(*) indicates the hub genes that are shared between the two types of metastases.

#### Functional analysis of protein network

**Fig 5:**
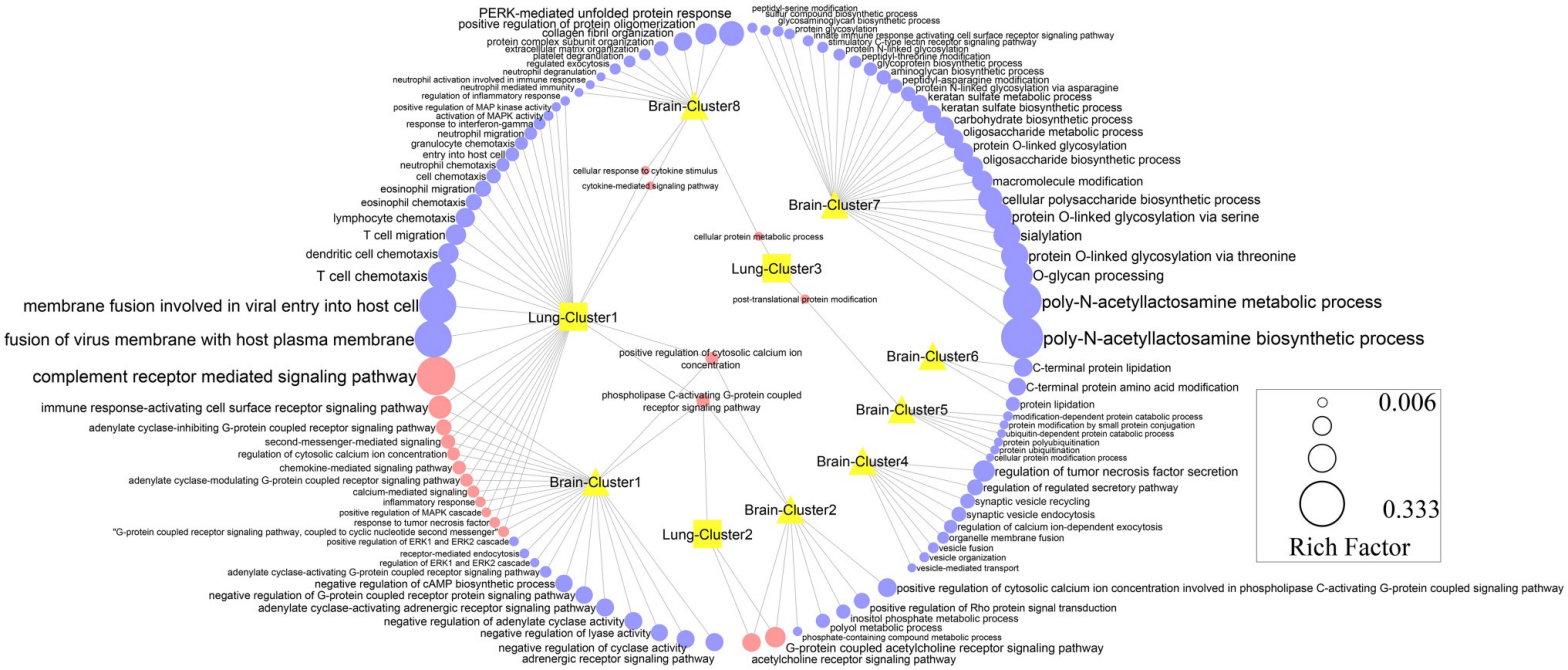
Biological process (BP) enrichment analysis for all function clusters of the brain and lung metastases network. The size of the circles indicates the gene numbers enriched in each BP. The adjusted P-value ≤0.05 was set as the threshold.

**Fig 6:**
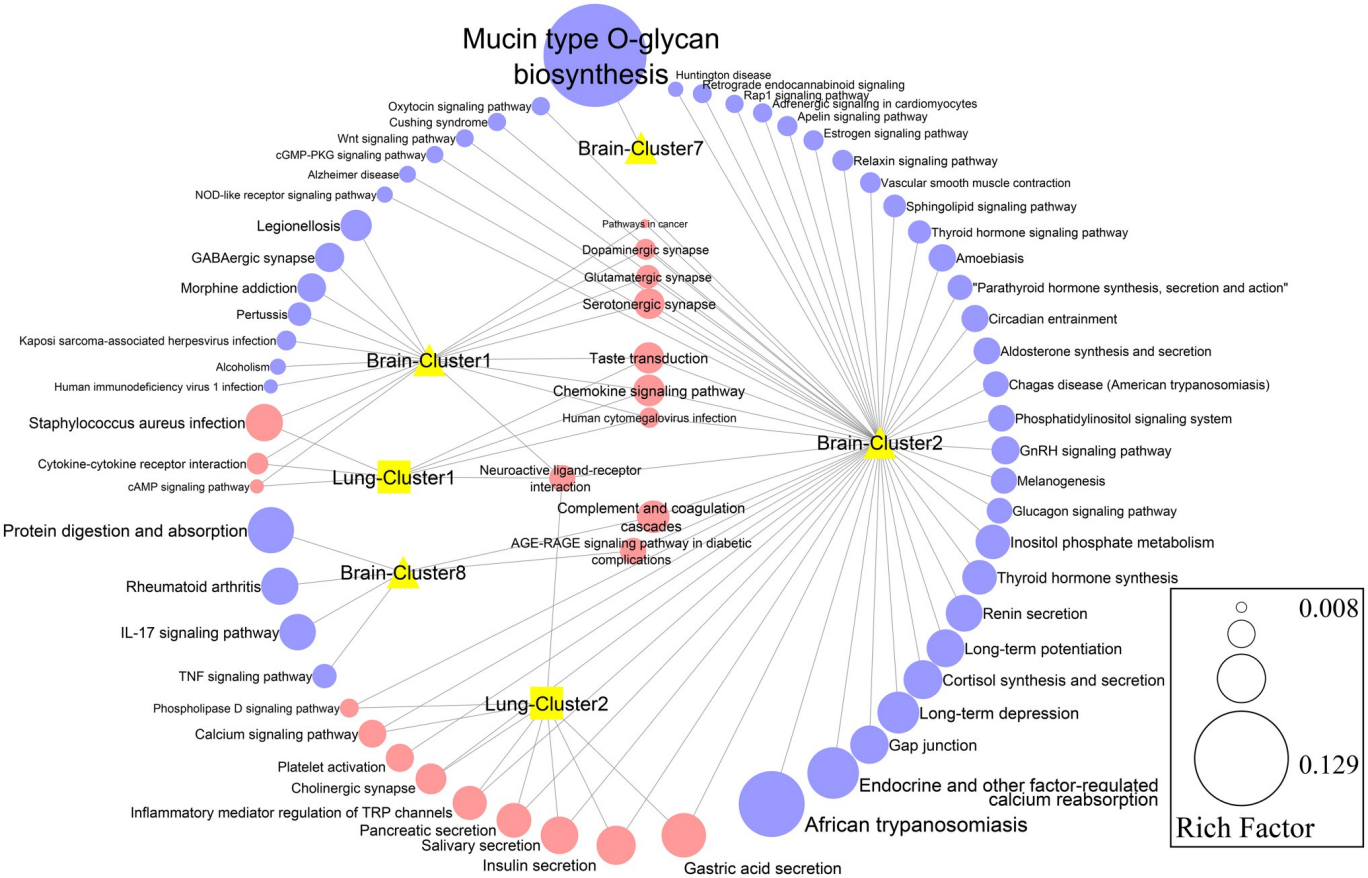
Kyoto Encyclopedia of Genes and Genomes (KEGG) enrichment analysis for all function clusters of the brain and lung metastases network. The size of the circles represents the number of genes enriched in each pathway. The adjusted P-value ≤0.05 was set as the cutoff.

The genes in the top eight subnet complexes were input into the Enrichr database for BP and KEGG pathway enrichment analysis (Figs 5 and 6 and S7 and S8 Tables). The most significantly enriched BPs in brain metastasis were the poly-N-acetyllactosamine biosynthetic process, O-glycan processing, and sialylation (Fig 5). The most outstanding KEGG pathways that were enriched in the brain metastasis were the mucin-type o-glycan biosynthesis pathway, endocrine and other factor-regulated calcium reabsorption pathways, and protein digestion and absorption pathway (Fig 6). Enrichment analysis showed that the most significant BPs in lung metastasis were T cell chemotaxis and dendritic cell chemotaxis (Fig 5). Complement receptor-mediated signalling pathway, acetylcholine receptor signalling pathway, and immune response-activating cell surface receptor signalling pathway were the most important shared BPs between the lung and brain metastases (Fig 5). Moreover, the gastric acid secretion pathway, insulin secretion pathway, and salivary secretion pathway were common KEGG pathways between the brain and lung metastases (Fig 6).

#### Protein complex analysis

**Fig 7:**
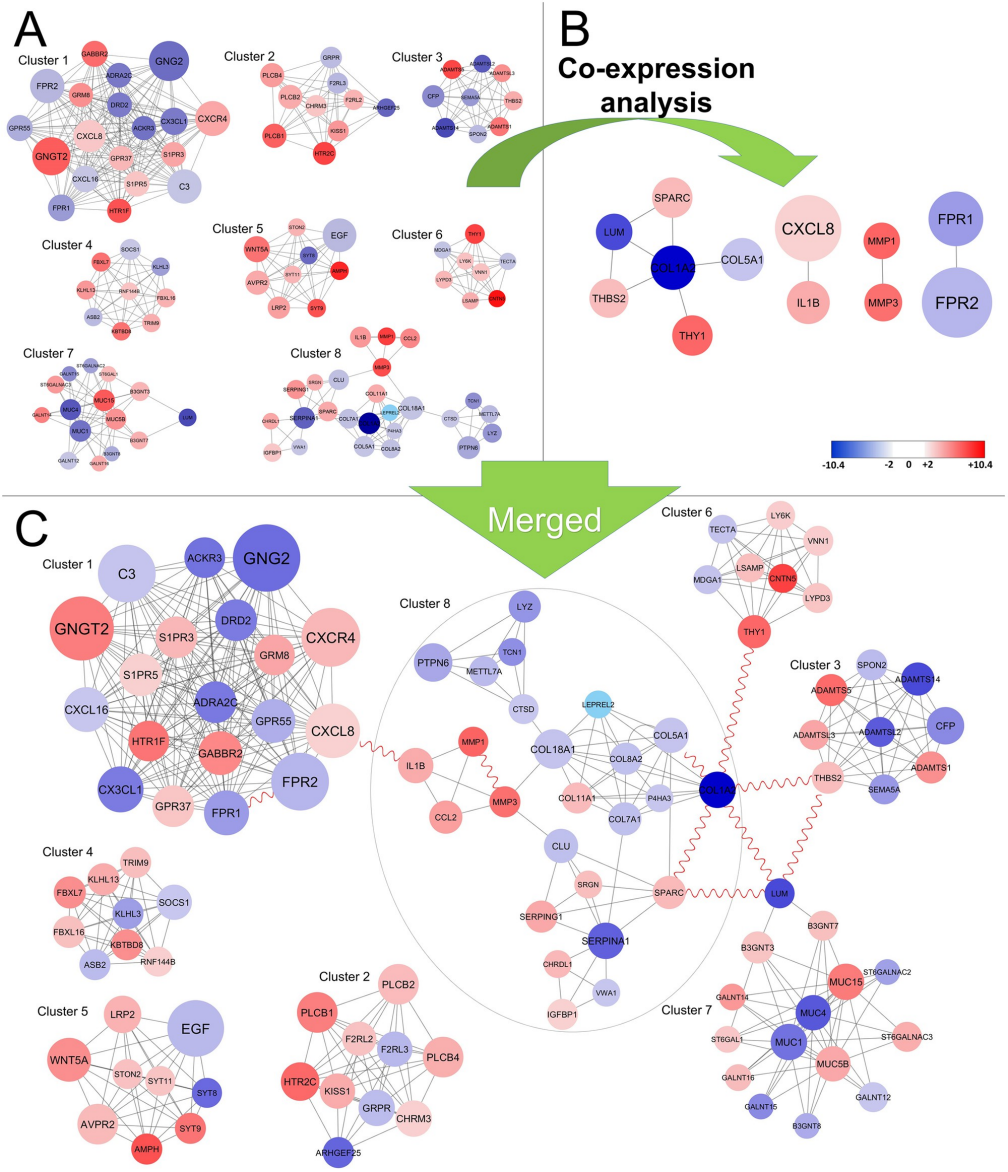
Subnetwork analysis from PPI network. A) Sub-network analysis of PPI network shows eight clusters in brain metastasis tumours. The colours represent differentially expressed values while the node size represents the count of interactions(degree) in the PPI network. B) Results of co-expression analysis obtained from eight clusters. C) The result of merging the clusters in A with co-expression in B to identify which clusters are co-regulated. To this end, co-expression genes were merged with eight clusters sub-networks to indicate a possibly co-regulated sub-network. Finally, co-expression edges showed with red sine waves indicate possible co-regulation between five clusters.

We used the MCODE plugin in the Cytoscape environment to analyze sub-network clustering. Eight clusters with a score ≥ 5 from the PPI network were screened for breast cancer brain metastatic cell line, BrM2 (Fig 7A and S6A Table). To indicate which clusters are co-regulated, we performed co-expression analysis on all eight clusters using minimum cut-off > = 4 (Fig 7A) and 12 proteins with 10 co-expression interactions were identified (Fig 7B). The eight clusters shown in Fig 7A were merged with the co-expression network in Fig 7B to illustrate co-expression connections between different clusters and predict which clusters were possibly co-regulated (Fig 7C). Hence, the 10 co-expressed edges between 12 nodes shown in Fig 7B reveal that out of eight total clusters, five are possibly co-regulated as seen in Fig 7C. Cluster 8 circled in Fig 7C includes 8 out of 10 co-expressed edges, indicating the importance of this cluster as it interacts with four other clusters (1, 3, 6, 7). The down expressed gene COL1A2 is shown to be responsible for five co-expressed interactions, three of which connect cluster 8 to other clusters (3, 6, 7). Besides, two further interactions of COL1A2 are inside cluster eight (Fig 7C). The LUM protein is co-expressed with COL1A2, SPARC from cluster eight and THBS2 from cluster three. Therefore, the LUM is a candidate that can co-regulate clusters 7 and 8 as well as clusters 3 and 8. Also, the THBS2 node represented two interactions with LUM and COL1A2 from cluster seven and eight, respectively. The THY1 and CXXL8 proteins are co-expressed with COL1A1 and IL1B, connecting cluster 8 to clusters 6 and 1, respectively. Clusters 6 and 8 are presumably co-regulated with THY1 and COL1A2 connection. Similarly, clusters 3 and 8 are co-regulated with THBS2 and COL1A2 interaction. Cluster 1 and 8 are also co-regulated with CXCL8 and IL1B interaction. Cluster 7 and 3 are also co-regulated by THBS2 and LUM interaction. Cluster 8 and 7 co-regulated with LUM-COL1A2 and LUM-SPARC interactions. Some interactions are co-expressed inside the cluster such as FPR1-FPR2, MMP3-MMP1, COL1A2-COL5A1, and COL1A2-SPARC interactions. These intracluster interactions maintain the connectivity of the network.

In the case of lung metastasis cell line from breast cancer, we identified four clusters with a score of ≥5 (S6B Table), revealing only one co-expression connection inside of cluster 1 (S1 Fig).

### Cancer dependency map (DepMap)

To evaluate the dependency of breast cancer MDA-MB-231 cell line on identified hub genes, gene knockout analysis by CRISPR and RNA interference (RNAi) was carried out. We examined the necessity of identified hub genes involved in brain and lung metastasis of breast cancer to determine the viability of the MDA-MB-231 cell line. In total, the results of gene knockout by RNAi and CRISPR revealed that 21 and 16 hub genes are essential for the MDA-MB-231 cell line, respectively. Eight genes CXCL8, C3, MMP9, GABBR2, WNT5A, S1PR5, CXCL16, and GNAO1 were affected by both CRISPR and RNAi gene knockout methods (S9 Table).

### Survival analysis of hub genes

**Fig 8:**
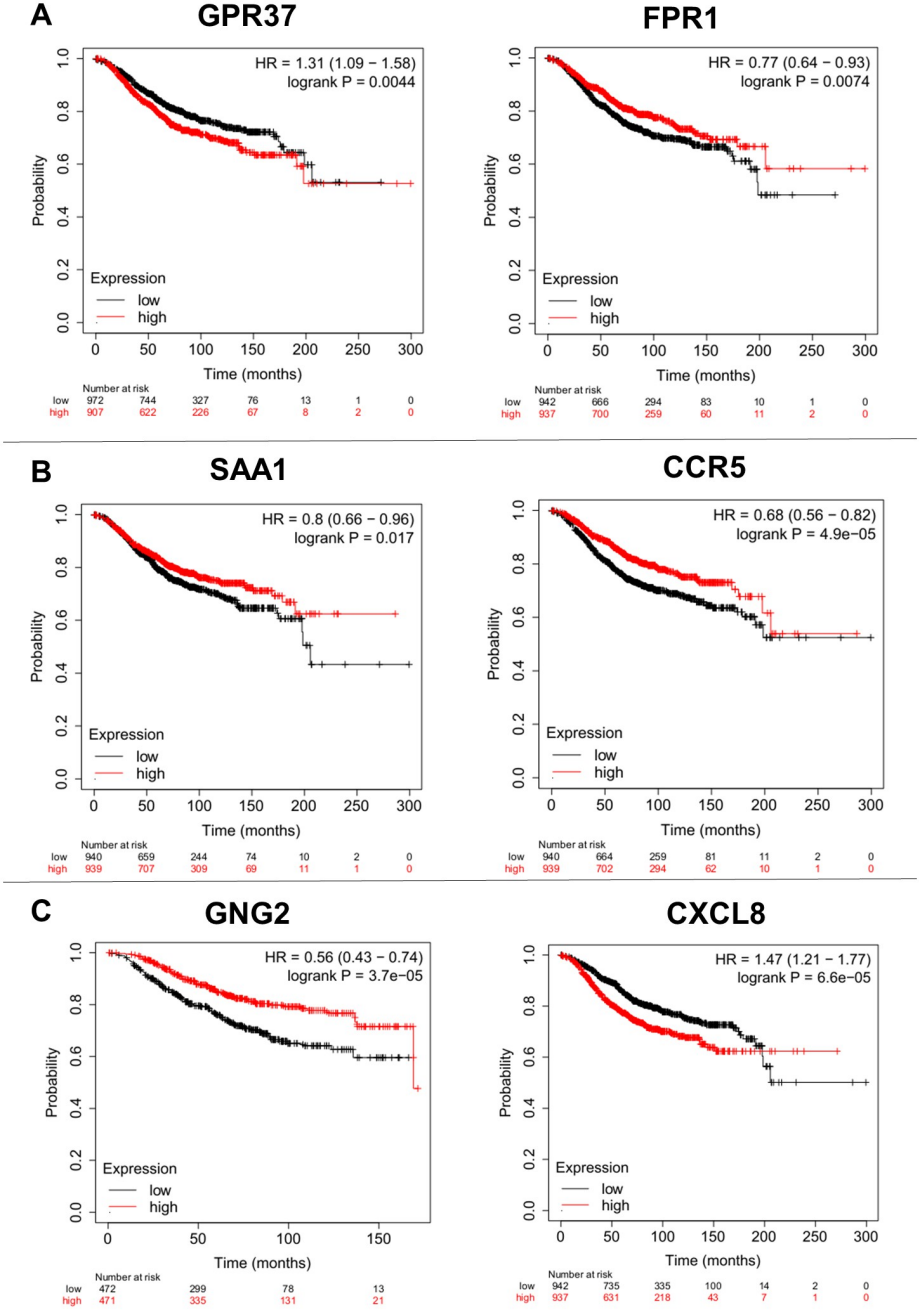
Prognostic values of four specific and two shared hub genes in the brain and lung metastases from breast cancer patients (Kaplan Meier-plotter database). (A) GPR37 and FPR1 which shared between brain and lung metastasis (B) SAA1 and CCR5 were specifically indicated in lung metastasis. (C) GNG2 and CXCL8 were specifically represented among brain metastasis samples. The red line implies the high-expression group and the black line represents the low-expression group. The high- and low-expression cohorts were divided by the median survival time.

We used overall survival analysis with the Kaplan-Meier plotter database to analyze the prognostic value of seven differentially expressed hub genes (FPR2, CXCR4, FPR1, CX3CL1, GPR37, GABBR2 and PLCB1) that were common in both the lung and brain metastases from breast cancer, but only GPR37 and FPR1 genes represent significantly (log-rank P = < 0.05) values (Fig 8A, S10 Table), as well as four specific hub genes of lung cell line (SAA1, CCR5, CCR3 and CXCL11) were significantly resulted with only two SAA1 and CCR5 genes (Fig 8B, S10 Table). Herein, the results of 27 specific hub genes of brain cell line were shown via seven significant values in survival analysis (Fig 8C, S10 Table).

Moreover, Up-regulation of GPR37 (HR 1.31, P = 0.0044) and CXCL8 (HR 1.47, P = 6.6e-05) and down-regulation of FPR1 (HR 0.64, P = 0.0074), SAA1 (HR 0.66, P = 0.0017), CCR5 (HR 0.56, P = 4.9e-05), GNG2 (HR 0.56, P = 0.3.7e-05), C3 (HR 0.67, P = 3.2e-05) and PTPN6 (HR 0.7, P = 0.00023) were significantly associated with an unfavorable overall survival in breast cancer patients (Fig 8, S2 Fig).

## Discussion

Metastasis is the main cause of mortality in breast cancer, with an overwhelming burden on the health system, especially in low and middle-income countries [41]. Therefore, a comprehensive investigation aiming reduction of the mortality rate must be conducted. In this study, we used bioinformatics approaches to examine the differences and similarities among gene expression profiles between LM2 and BrM2 metastases cell line obtained from breast cancer patients. A panel of 981 and 662 DEGs were identified to have an association with breast cancer metastasis to the brain and lungs, respectively (Fig 1 and S3 and S4 Tables). Among these, we identified seven hub genes (FPR2, CXCR4, FPR1, CX3CL1, GPR37, GABBR2 and PLCB1) that overlapped between the brain and lung metastases (Table 1). Survival analysis showed that the over-expression of GPR37 and the down-regulation of FPR1 were associated with poor overall survival in breast cancer patients (Fig 8), indicating that these hub genes may potentially be a driver for breast cancer development.

PLCB is an enzyme that plays a key role in the cell cycle and proliferation, both of which are important in tumour initiation and development [42]. Sengelaub et al. demonstrated the up-regulation of PLCB1 in extremely metastatic breast cancer cells [43]. Formyl-peptide receptors (FPRs) belong to the chemotactic G-protein-coupled receptor (GPCRs) family have an important role in inflammation, immune responses, and cancer progression [44]. Vacchelli et al. reported that poor patient outcome and metastasis-free in breast and colorectal cancer patients who underwent adjuvant chemotherapy was linked to a loss-of-function allele of the gene that encodes FPR1 [45]. Moreover, the ALX/FPR2 axis, a main stop signal of inflammation, was shown to be down-regulated in MDA-MB-231 cancer cell lines [46].

Previous studies have revealed that the chemokine CXCL12 can bind to the CXCR4 receptor and lead to cell chemotaxis, proliferation, and gene transcription. CXCR4 overexpression in cancer cells is associated with tumour growth, angiogenesis, and metastasis [47]. Interestingly, we found that the CXCR4 gene had a different pattern of expression in the BrM2 cell line compared to LM2 (Table 1). This gene was over-expressed in the brain metastatic cell line, but down-regulated in the LM2 cell line. Nobutani et al. demonstrated that the expression level of CXCR4 was highly dependent on the change of the tumour environment. They showed that the expression level of CXCR4 was attenuated in lung metastasis from breast cancer compared to cancer cells in orthotopic tumours because the orthotopic tumours were subjected to harsher stress-promoting factors (e.g. hypoxia and shortage of nutrients) than the metastatic cells in the lungs [48]. Nevertheless, it has been shown that CXCR4 expression was agumented in both lung and brain metastases induced by breast cancer [49]. Therefore, the difference in the tumour environment of the lungs and brain may be the possible reason for the different expression level of CXCR4. Taken together, we assume that the different patterns of CXCR4 expression determine which tissue is to be metastasized, suggesting that CXCR4 could be a prognostic biomarker for metastasis classification in breast cancer.

G protein-coupled receptors (GPRs) are located on the cell’s surface and are involved in many biological and physiological processes. Cancer cells dysregulate their normal physiological functions, leading to tumour growth and metastasis [50]. Our analysis demonstrated that GPR37, as a hub gene, was up-regulated in both brain and lung metastatic breast cancer cell lines. In line with this, Kubler et al. showed that GPR37 is a receptor for prosaptide ligand and over-expressed in breast cancer tissues [51]. Considering the aforementioned reports, these genes might be potential biomarkers for the diagnosis of breast cancer and its metastasis.

In addition, we identified 34 hub genes for the brain metastatic breast cancer cell line. Among them, 27 hub genes were specific in the BrM2 cell line: GNG2, GNGT2, C3, EGF, CXCL8, MMP9, CXCL16, GPR55, WNT5A, S1PR5, ADRA2C, DRD2, TIMP1, S1PR3, ACKR3, PLCB2, AVPR2, PLCB4, GRM8, HTR1F, COL18A1, PTPN6, GNAO1, ITGB2, HTR2C, CFP, LRP2 (Table 1). Among these genes, we conducted a more thorough investigation of the GNG2, CXCL8, C3, and PTPN6 genes, because they were associated with poor patients outcome (Fig 8, S2 Fig).

GNG2 is involved in the structure of the heterotrimeric G protein [52] and affects the development and localization of metastases [53]. Although previous studies confirmed the down-expression of GNG2 in malignant melanoma [54] and pancreatic ductal adenocarcinoma (PDAC) [55] cancers, there has been no discussion so far about the role of GNG2 in breast cancer and its metastasis to the brain. Interestingly, we found that the down-expression of GNG2 is associated with breast cancer metastasis to the brain. This gene can be of interest to clinical researchers due to the provided therapeutic insights.

Brysse et al. reported that the mRNA expression of CXCL8 was decreased upon siRNA transfection against ZO-1 in invasive breast cancer cells, but not in non-invasive breast cancer cells, suggesting that the CXCL8 plays a key role in breast cancer progression [56]. Complement component C3 (C3) is a protein that plays an important role in innate immunity. Dowling et al. demonstrated that this gene is among the best candidate biomarkers in breast cancer [57], as we discussed in this research. Several studies have shown that the DNA methylation-mediated down-regulation of protein tyrosin phosphatase non-receptor type 6 (PTPN6) was linked with the progression of esophageal squamous cell carcinoma [58] and gastric cancer [59]. Therefore, it seems down-regulation of this gene could potentially be a prognostic biomarker in breast cancer brain metastasis as well.

We also identified 11 hub genes related to the LM2 cell line, of which four genes including SAA1, CCR3, CCR5, and CXCL11 were specific to lungs metastasis. Among them, down-regulation of SAA1 and CCR5 was associated with poor overall survival in breast cancer patients (Fig 8). In an effort, Ni et al. showed the prognostic value of SAA in patients with hepatocellular carcinoma [60]. Moreover, Stange et al. identified 15 genes including SAA1 that were down-regulated in colorectal metastasis to the liver [61]. We thus propose that the SAA1 gene may potentially be a prognostic candidate for breast cancer metastasis to the lungs. Previous studies have reported that the CCR5 over-expression reinforced the CRC metastasis to distant organs [62, 63]. However, the CCR5 status in breast cancer lung metastasis has not been reported yet. We, therefore, propose that the down-regulation of CCR5 could be a diagnostic biomarker for breast cancer progression to the lungs.

Gene knockout analysis using RNAi on the hub genes was shown to be associated with brain metastasis in the MDA-MB-231 cell line in which the GNGT2 gene has a major role in decreased viability. By contrast, among the common hub genes between brain and lung metastases, only RNAi knockout of the FPR2 gene affected the cell line viability. Overall, gene knockout results show that most of the identified hub genes could be critical for the MDA-MB-231 cell line (S9 Table).

In the current study, eight sub-networks were identified by the MCODE plugin for brain metastasis and four sub-networks for lung metastasis from breast cancer (S6 Table). We postulate that cluster 8 with eight co-expression connections to clusters 1, 3, 6, and 7 may be linked to each other and act as a system in brain tumour metastasis. (Fig 7C). We demonstrated that COL1A2 is an important gene with five co-expression interactions with the other three clusters. Evaluation of differentially expressed genes between primary breast cancer and brain metastatic tissues showed that the COL1A2 gene is down-regulated in brain metastatic compared with primary pairs, indicating that this gene is critical to breast cancer progression to the brain [64]. Lumican, also known as LUM, is another significant co-regulated gene that connects cluster 7 to clusters 8 and 3 with three co-expression interactions (Fig 7C). Karamanou et al. have reported that the invasion and proliferation index of the MDA-MB-231 cell line is inversely associated with the down-regulation of the LUM gene, supporting our findings that down-regulation of this gene could be an essential factor involved in breast cancer brain metastasis [65].

THBS2 gene is also responsible for a co-regulation between cluster 3 and clusters 7–8. Gene expression analysis between ductal carcinoma *in situ* (DCIS) and invasive breast carcinoma (IBC) tissues has indicated that the THBS2 gene is up-regulated during the transition between non-invasive to invasive breast cancer [66]. Furthermore, four other candidate genes SPARC, THY1, IL1B and CXCL8 are reported in this study as the key regulatory genes which can modulate co-regulatory between different clusters in the PPI network of the brain metastasis tumours from breast cancers (Fig 7C). Accordingly, special attention to these genes in translational oncology settings may lead to the discovery of novel important biomarkers in the spread pathway of breast cancer to the brain.

Sub-network analysis significantly indicated that the genes involved in clusters 1 and 2 of the PPI for brain metastasis, as well as the results from GO and KEGG, were largely similar to those in clusters 1 and 2 of PPI analysis of the lung metastasis (Figs 5 and 6 and S6 and S7 Tables), which indicates that these two clusters are significantly shared between brain and lung metastases. The most significantly shared BP between brain and lung metastases was the complement receptor-mediated signalling pathway (Fig 5). Vadrevu et al. showed that the complement anaphylatoxin C5a receptor (C5aR) promotes lungs metastasis of breast cancer in a pre-clinical mouse model of breast cancer by abolishing CD8^+^ and CD4^+^ T-cell responses [67]. Therefore, complement receptors can serve as potential targets of immunotherapy drugs in breast cancer metastasis.

However, some BPs were identified as unique BPs in either the brain or lung metastasis. The most important BP which dysregulated only in brain metastasis was the poly-*N*-acetyllactosamine biosynthetic process (Fig 5). It has been reported that cell-cell interaction and metastatic potential of tumour cells are facilitated by stably synthesizing the poly-*N*-acetyllactosamine chain on the extracellular side of cancer cells. In the breast cancer cells, poly-*N*-acetyllactosamine branching promotes further glycan modification, increasing its metastatic potential significantly [68]. These data suggest that this BP may have a specific driver function in brain metastasis from breast cancer.

In addition, T cell chemotaxis was the main BP that was correlated with lung metastasis from breast cancer (Fig 5). Olkhanud et al. showed that breast cancer lung metastasis is mediated by chemokine receptor-induced chemotaxis, but this process is not adequate to promote metastasis due to the elimination of tumour cells in the lung by natural killer (NK) cells. They explained that lung metastasis needed chemokine receptor regulatory T cells (Treg) to eliminate NK cells; thereby, tumour cells become highly colonized in the lungs [69]. All in all, our results support the previous report, demonstrating the involvement of this BP in the progression of breast cancer.

KEGG pathway analysis of the DEGs from all sub-networks revealed that the most prominent shared pathways between the lung and brain metastases were the gastric acid secretion and insulin secretion signalling pathways (Fig 6). Numerous pieces of evidence are supporting the role of gastrin-releasing peptide (GRP) in the initiation and progression of breast cancer. Miyazaki et al. have demonstrated that the growth of MDA-MB-231 human breast cancer xenografts in nude mice is inhibited by utilizing antagonist molecules such as RC-3940-II and RC-3095 that target GRP growth factor [70]. Moreover, the effect of gastrin-releasing peptides in lymph node metastasis of breast cancer has been investigated [71]. These data indicate that the gastric secretion track may be considered as a useful way of diagnosing brain and lung metastasis from breast cancer. Additionally, it has been shown that high levels of insulin caused by abnormal insulin secretion kinetics are positively correlated with breast cancer progression [72].

Fig 6 shows that the most significant pathways associated with brain metastasis breast cancer were mucin-type O-glycan biosynthesis, and endocrine and other factor-regulated calcium reabsorption. Mayoral et al. have demonstrated that biosynthesis of mucin-type O-glycans is dysregulated in breast cancer metastasis to the brain [73]; which is an agreement with our observation in this study. It has been demonstrated that in the MDA-MB-231 breast cancer cell line, the secretion of PTH-related peptide (PTHrP) is increased in response to elevated extracellular calcium. Subsequently, Ca^2+^-sensing receptor (CaR) is over-expressed in this cell line, suggesting that reabsorption of calcium by the MDA-MB-231 cell line is of importance for its further growth [74]. Collectively, we predict that these two pathways determine the invasion and metastasis of breast cancer to specific target tissues.

Several studies in the literature have used gene expression profiling to identify differentially expressed genes and functional pathways in brain and lung metastases from breast cancer. For instance, Massaque et al. identified 18 differentially expressed genes, including IL13Ra2, that mediate breast cancer metastasis to the lungs. These findings support our observations (S3 and S4 Tables). They reported that the IL13Ra2 expression level was highly associated with aggressive lung metastatic populations [15]. Engin et al. have identified seven hub genes, including MMP1, that were associated with brain metastasis from breast cancer and seven key nodes, including CXCR4 and MMP1, for lung metastasis from breast cancer [17]. As shown in Table 1, we also identified CXCR4 as a hub gene in both metastases. Moreover, we identified that the MMP1 gene is over-expressed in both metastases cell lines (S3 and S4 Tables), as a previous study reported. Tang et al. used PPI network analysis and hub gene identification approaches to identify 10 hub genes, including VCAM1, that were associated with brain metastasis from breast cancer [75]. It has been demonstrated that down-expression of the VCAM1 gene supports tumour growth in brain metastasis from breast cancer [16], which is in agreement with our findings (S3 Table).

Zhang et al. developed a predictive computational method named DryNetMC to distinguish gene regulatory networks of drug-resistance and drug-sensitive glioma cell lines [76]. They utilized time-course RNA-seq data from glioma cells to identify key genes as predictors for drug-sensitivities of some glioma cell lines. The top-ranked genes that they identified to be associated with targeted therapy response were KIF2C, CCNA2, NDC80, KIF11, KIF23 [76]. Zaman et al. integrated the exome-sequencing data (mutation and copy number variations) with functional RNAi screening data aiming at identifying subtype-specific signalling networks for breast cancer cell lines. Based on their findings, AKT1, mTOR, MET, MDM2, HSP90AA1, RAF1, SFN, and ESR1 were the most significant luminal-specific drug targets, whereas TGF-β, IGF1R, MAPK3, GRB2, SRC, TUBB, JAK2, and EGFR were introduced as powerful basal-specific biomarkers [77]. A plethora of studies discussed the pivotal role of somatic mutations in tumorigenesis and paid less attention to the role of germline variations. However, Milanese et al. provided a new notion into the predictive role of germline variants associated with patients ‘outcome in ER^+^ breast cancer patients. Using the eTumorMetastasis method, they found that patients with tumour recurrence harbour a higher rate of germline variants; more specifically in leukocyte genes including T cells and APCs, indicating that immune response impairing may be the cause of tumour relapse in breast cancer patients [78]. In the present study, we comprehensively analyzed DEGs that may provide new insights regarding genes that mediate brain and lung metastases from breast cancer.

## Conclusions

In conclusion, we have some candidates into three levels of content. During the metastasis process for the first time, we identified two candidate hub genes FPR1 and GPR37 were shared between brain and lung tumours that have likely been associated with the metastases process. To the pathways analysis, we showed in the present study two pathways that were shared between brain and lung metastasis tumours. In the second level, we try to define specific features from each tumour separately to this end, we are indicating four hub genes only in the brain tumours that included GNG2, CXCL8, C3, and PTPN6 with also report three pathways poly-*N*-acetyllactosamine biosynthetic process, mucin-type O-glycan biosynthesis pathway, and calcium reabsorption pathway specifically for the brain metastasis tumours. Furthermore, to the lung-specific tumours, two hub genes were reported as a specific prognosis biomarker SAA1 and CCR5 with also T cell chemotaxis pathway This finding indicates that some pathways specifically determine the organ breast cancer metastasizes to. The benefits of the specific features in the second level of this study are around the potential diagnostic and prognostic and therapeutic targets for breast cancers tumours that are metastasized into brain and lung tissues, respectively. To the third level. we have reported five co-regulated clusters via seven important co-expression genes (COL1A2, LUM, SPARC, THBS2, IL1B, CXCL8, THY1) interacting between five clusters of the brain tumours. These seven genes (especially COL1A2, LUM, and THBS2 genes) can be utilized as therapeutic targets to inhibit connection between each clusters in breast cancer metastasis to the brain. Thoroughly, further studies are needed to validate these observations and determine their clinical utility in the therapeutic management of breast cancer metastasis. Taken together, by using RNA sequencing analysis, we have explored breast cancer metastasis signatures that may pave the way for better metastatic diagnosis and tissue-specific genes to tissue-specific therapy for breast cancer metastasis.

## Supporting information

S1 Fig The four clusters of lung protein-protein interactions. Sub-network analysis from the PPI network showed four clusters in lung metastasis tumours. The colours represent differentially expressed genes and also the sizes of nodes were indicating the count of interactions (degree) in the PPI network.
(JPG)

S2 Fig The survival analysis of the brain hub genes. Prognostic values of two specific hub genes in the brain metastases from breast cancer patients (Kaplan Meier-plotter database). **(A)** C3 and **(B)** PTPN6 were specifically dysregulated in brain metastasis. The red line implies the high-expression group and the black line represents the low-expression group. The high- and low-expression cohorts were divided by the median survival time.
(TIF)

S1 Table Samples of data analyzed. Samples information that is used in this study is represented. Column cell line indicated cell line of samples and also column two showed the type of cell lines. In order, column three indicates an explanation of cell line, since that column four showed the count of each replicate of the sample. By the sequence, GEO accessions are shown in the final column.
(DOCX)

S2 Table Indicating whole 17,325 genes expression were used in this study. (TXT)

S3 Table The 981 DEGs in brain metastasis. (TXT)

S4 Table The 662 DEGs in lung metastasis. (TXT)

S5 Table Common DEGs between brain and lung metastasis cell line from breast cancer. Which are represented A) Common up-regulated genes between brain and lung metastasis cell line from breast cancer. B) Common down-regulated genes between brain and lung metastasis cell line from breast cancer.
(DOCX)

S6 Table Ranked clusters of PPI networks analyzed by MCODE. Which are represented (A) Clusters obtained from brain metastatic breast cancer PPI network. (B) Clusters obtained from lung metastatic breast cancer PPI network.
(DOCX)

S7 Table Biological process terms for protein members of the clusters obtained from brain and lung DEG network. (XLSX)

S8 Table KEGG pathways for protein members of the clusters obtained from brain and lung DEG network. (XLSX)

S9 Table CRISPR and RNAi represent the gene knockout methods. Which are implemented on the identified hub genes in the MDA-MB-231 cell line. (*) indicates the genes that are shared between Brain and Lung tumour metastases.
(DOCX)

S10 Table The survival analysis to three types of hub genes. The Type column represents where genes are differentially expressed as specific which is expressed in the brain and doesn’t express in Lung and which are differentially expressed in both Lung and Brain tumours that metastasis from Breast cancer. The Name column showed the Gene Symbol of each gene and HR the column represents Hazard Ratio value and also log-rank P column showed each significant (log-rank P = < 0.05) values.
(DOCX)
