# Supplementary figures and images for: Comparative analysis of protein-protein interaction networks in metastatic breast cancer
Source: PLoS One. 2022 Jan 19;17(1):e0260584. doi: 10.1371/journal.pone.0260584 (PMC8769308; doi:10.1371/journal.pone.0260584)

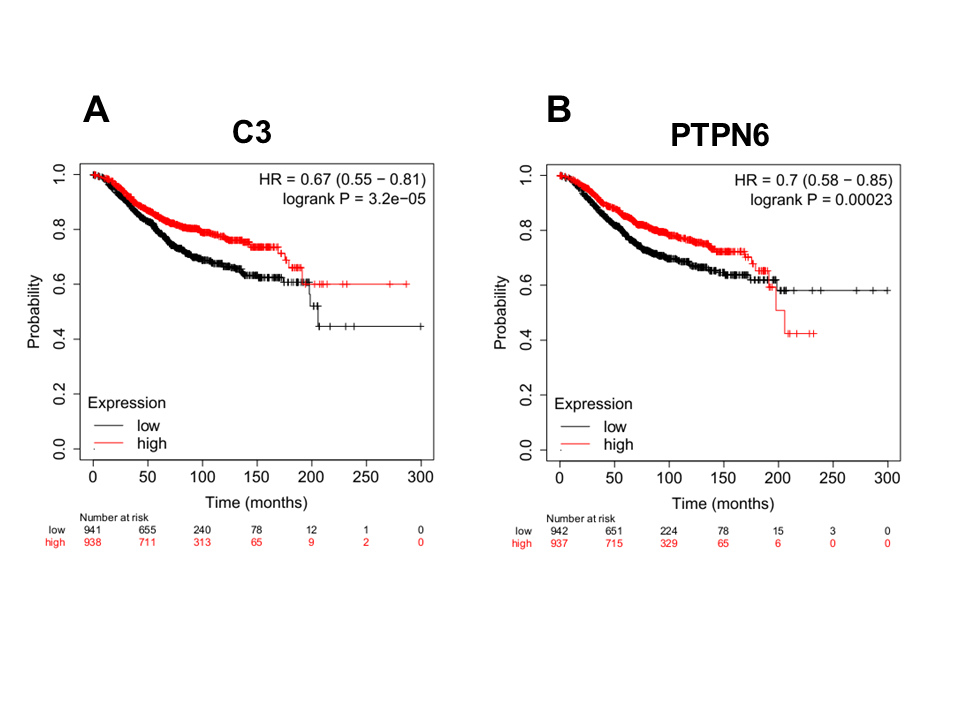

Supplement: S2 Fig — Prognostic values of two specific hub genes in the brain metastases from breast cancer patients (Kaplan Meier-plotter database). (A) C3 and (B) PTPN6 were specifically dysregulated in brain metastasis. The red line implies the high-expression group and the black line represents the low-expression group. The high- and low-expression cohorts were divided by the median survival time. (TIF) [file pone.0260584.s002.tif]
