# Supplementary material for: Comparative analysis of protein-protein interaction networks in metastatic breast cancer
Source: PLoS One. 2022 Jan 19;17(1):e0260584. doi: 10.1371/journal.pone.0260584 (PMC8769308; doi:10.1371/journal.pone.0260584)
Supplement: S1 Table — Samples information that is used in this study is represented. Column cell line indicated cell line of samples and also column two showed the type of cell lines. In order, column three indicates an explanation of cell line, since that column four showed the count of each replicate of the sample. By the sequence, GEO accessions are shown in the final column. (DOCX) [file pone.0260584.s003.docx]

| Cell lines | Type | Description | Replicate | GEO accession |
| --- | --- | --- | --- | --- |
| MDA-MB-231 | Parental | A model for late-stage of breast cancer | 3 | GSM4100708  GSM4100709  GSM4100710 |
| MDA231-BrM2-831 | Sub population | Brain metastatic model | 3 | GSM4100702  GSM4100703  GSM4100704 |
| MDA231-LM2-4175 | Sub population | Lung metastatic model | 3 | GSM4100705  GSM4100706  GSM4100707 |

**S1. Table. Samples of data analyzed**

Samples information that is used in this study is represented. Column cell line indicated cell line of samples and also column two showed the type of cell lines. In order, column three indicates the explanation of cell line, since that column four showed the count of each replicate of a sample. By the sequence, GEO accessions are shown in the final column.
