# Supplementary material for: Comparative analysis of protein-protein interaction networks in metastatic breast cancer
Source: PLoS One. 2022 Jan 19;17(1):e0260584. doi: 10.1371/journal.pone.0260584 (PMC8769308; doi:10.1371/journal.pone.0260584)
Supplement: S5 Table — Which are represented A) Common up-regulated genes between brain and lung metastasis cell line from breast cancer. B) Common down-regulated genes between brain and lung metastasis cell line from breast cancer. (DOCX) [file pone.0260584.s007.docx]

**S5 Table. Common DEGs between brain and lung metastasis cell line from breast cancer.**

Which are represented A) Common up-regulated genes between brain and lung metastasis cell line from breast cancer. B) Common down-regulated genes between brain and lung metastasis cell line from breast cancer.

| A. Common up-regulated genes between brain and lung metastasis | | | | |  | B. Common down-regulated genes between brain and lung metastasis | | | | |
| --- | --- | --- | --- | --- | --- | --- | --- | --- | --- | --- |
| symbol | LogFC for Brain | adjp for Brain | LogFC for Lung | adjp for Lung |  | symbol | LogFC for Brain | adjp for Brain | LogFC for Lung | adjp for Lung |
| ADAM23 | 3.132225 | 7.57E-08 | 2.935711 | 7.65E-07 |  | **ABCA1** | -2.90788 | 9.38E-27 | -2.30379 | 3.60E-17 |
| ADD2 | 6.076275 | 5.03E-21 | 5.155868 | 6.13E-15 |  | **ACTBL2** | -6.1058 | 0.000443 | -6.4437 | 0.000243 |
| ADM | 2.708039 | 2.12E-09 | 2.746703 | 1.59E-09 |  | **ADAM28** | -6.92571 | 3.50E-14 | -3.64043 | 1.86E-08 |
| AHRR | 2.793565 | 1.80E-13 | 2.268674 | 8.31E-09 |  | **ADGRF1** | -7.57341 | 1.90E-66 | -2.29312 | 6.46E-16 |
| AIF1L | 2.452073 | 1.46E-11 | 2.986453 | 5.53E-17 |  | **AKR1C2** | -2.29486 | 3.20E-07 | -2.22438 | 3.80E-07 |
| AMPH | 8.781361 | 2.77E-62 | 6.953583 | 1.09E-38 |  | **ALG1L7P** | -2.63784 | 0.016212 | -2.53121 | 0.017155 |
| ANO2 | 3.856606 | 1.60E-20 | 2.998237 | 3.29E-12 |  | **ALOX5** | -3.15354 | 1.15E-08 | -5.30428 | 7.86E-15 |
| ANO3 | 7.334278 | 1.01E-06 | 6.467295 | 3.80E-05 |  | **ANGPTL2** | -2.0733 | 5.88E-06 | -2.21129 | 1.35E-06 |
| APBB1IP | 2.398266 | 8.35E-21 | 2.211764 | 1.32E-17 |  | **ANXA8L1** | -3.17749 | 9.67E-11 | -2.66482 | 3.57E-08 |
| AR | 3.695128 | 1.28E-43 | 4.428323 | 9.94E-64 |  | **APCS** | -6.7636 | 1.58E-05 | -7.1015 | 6.64E-06 |
| BCL2A1 | 6.323501 | 5.79E-10 | 3.982883 | 0.000617 |  | **AQP1** | -2.85934 | 1.56E-06 | -2.36292 | 0.000128 |
| BEX5 | 7.161406 | 8.34E-07 | 4.379136 | 0.012538 |  | **AREG** | -4.13632 | 2.30E-47 | -3.66765 | 1.76E-39 |
| BMP2 | 2.30155 | 9.36E-05 | 2.761044 | 7.86E-07 |  | **ARSI** | -4.06892 | 2.01E-12 | -2.28452 | 0.000176 |
| C20orf197 | 2.688322 | 0.009486 | 3.373807 | 0.000452 |  | **ASTN1** | -5.18312 | 0.002539 | -4.58279 | 0.004147 |
| CASC9 | 3.868838 | 0.039268 | 5.489319 | 0.000903 |  | **ATP1A2** | -3.39048 | 0.027952 | -6.07614 | 0.000422 |
| CASP1 | 2.350555 | 4.08E-05 | 3.517758 | 2.58E-11 |  | **ATP2B2** | -6.78755 | 6.36E-08 | -7.96148 | 2.69E-08 |
| CD28 | 2.561037 | 0.001169 | 2.342303 | 0.005046 |  | **B3GNT8** | -3.15643 | 0.000259 | -3.43661 | 6.13E-05 |
| CHRM3 | 2.107702 | 2.70E-08 | 2.767554 | 2.10E-14 |  | **C11orf44** | -2.10383 | 1.58E-05 | -2.63617 | 6.87E-08 |
| CHST9 | 2.074126 | 0.045108 | 2.376931 | 0.017593 |  | **C11orf86** | -4.46329 | 0.000174 | -2.62384 | 0.019415 |
| CNN1 | 3.683202 | 0.001577 | 4.064369 | 0.000413 |  | **C1orf115** | -7.48335 | 2.34E-07 | -6.85945 | 4.18E-06 |
| CNTN1 | 3.092104 | 0.031475 | 4.020111 | 0.002451 |  | **C2** | -4.43999 | 5.62E-06 | -2.52923 | 0.000248 |
| CNTN5 | 9.617241 | 2.27E-12 | 5.115687 | 0.001811 |  | **C6orf223** | -7.37267 | 1.84E-12 | -4.82129 | 1.82E-15 |
| CYP24A1 | 3.042646 | 9.15E-20 | 2.170923 | 7.08E-10 |  | **C7orf57** | -3.8286 | 0.000701 | -3.5512 | 0.000526 |
| DGKG | 7.231801 | 2.85E-07 | 8.932162 | 6.12E-11 |  | **CAMK2A** | -5.59 | 0.000353 | -2.41788 | 0.008885 |
| DOCK10 | 2.708094 | 0.000559 | 2.883553 | 0.000265 |  | **CCDC3** | -2.10018 | 4.36E-05 | -2.93348 | 3.04E-09 |
| DOK6 | 4.111327 | 0.008237 | 4.657188 | 0.002247 |  | **CCDC74A** | -2.78571 | 3.12E-10 | -2.08552 | 8.59E-07 |
| DTNA | 2.523179 | 9.67E-11 | 2.094618 | 2.31E-07 |  | **CFP** | -4.60886 | 1.31E-13 | -3.95253 | 5.74E-13 |
| DUSP1 | 2.277435 | 4.87E-06 | 3.285359 | 5.04E-12 |  | **CHI3L2** | -3.94352 | 0.003569 | -3.62568 | 0.003489 |
| DUXAP8 | 2.662433 | 2.42E-16 | 3.02914 | 3.18E-21 |  | **CLDN2** | -4.10687 | 3.47E-25 | -3.0098 | 1.36E-15 |
| DUXAP9 | 4.052169 | 2.42E-21 | 3.313811 | 4.02E-14 |  | **CLDN4** | -2.19503 | 4.18E-05 | -3.98275 | 1.50E-15 |
| EAF2 | 3.819774 | 0.000154 | 2.822047 | 0.015742 |  | **CLIC3** | -5.5416 | 1.95E-11 | -4.31402 | 1.67E-10 |
| ELMOD1 | 3.277262 | 2.18E-05 | 2.880998 | 0.000416 |  | **CMPK2** | -4.01256 | 0.003201 | -2.56182 | 0.025491 |
| EMB | 2.538669 | 0.000522 | 2.094559 | 0.009274 |  | **CNGA1** | -2.08462 | 0.000156 | -2.49673 | 4.70E-06 |
| ENG | 4.253262 | 1.31E-29 | 4.267744 | 1.03E-29 |  | **CNKSR1** | -2.40283 | 5.79E-07 | -3.72963 | 4.86E-13 |
| ENPP2 | 2.610542 | 5.51E-07 | 2.072534 | 0.000222 |  | **COL1A2** | -10.4035 | 1.51E-15 | -2.23443 | 2.18E-11 |
| ESM1 | 7.166687 | 5.08E-05 | 7.048927 | 9.85E-05 |  | **CPNE7** | -3.46657 | 2.24E-09 | -2.20539 | 0.000112 |
| FAM155A | 2.912625 | 1.16E-06 | 2.915493 | 1.49E-06 |  | **CREG2** | -2.95806 | 0.002007 | -3.42303 | 0.000433 |
| FBLN5 | 3.617595 | 5.09E-09 | 3.949361 | 9.91E-11 |  | **CRTAM** | -5.36619 | 3.82E-11 | -4.12944 | 1.90E-11 |
| FBXL7 | 5.585802 | 9.17E-26 | 4.678437 | 8.55E-18 |  | **CST1** | -2.77427 | 8.50E-06 | -6.33174 | 1.19E-26 |
| FGF13 | 3.698705 | 2.33E-05 | 4.624002 | 2.69E-08 |  | **CST4** | -2.41539 | 0.002412 | -4.9005 | 9.14E-12 |
| FKBP10 | 5.830634 | 1.05E-25 | 7.857078 | 6.01E-47 |  | **CST6** | -3.71105 | 4.98E-14 | -3.07208 | 3.01E-11 |
| FLT1 | 6.734086 | 2.33E-06 | 4.693698 | 0.00414 |  | **CX3CL1** | -5.20465 | 8.11E-19 | -3.43546 | 6.68E-12 |
| FOXA1 | 6.909145 | 1.24E-06 | 5.200732 | 0.000962 |  | **CXCL16** | -2.08095 | 0.022498 | -2.74175 | 0.00184 |
| FZD8 | 2.499559 | 9.38E-09 | 2.016319 | 1.11E-05 |  | **CYGB** | -2.55387 | 0.000922 | -2.53948 | 0.001122 |
| GABBR2 | 5.870403 | 3.65E-17 | 4.39058 | 2.20E-09 |  | **CYP1B1** | -2.90494 | 9.72E-11 | -2.41258 | 2.11E-07 |
| GALNT14 | 4.966859 | 8.70E-25 | 3.984314 | 9.24E-16 |  | **DGCR5** | -4.49497 | 3.21E-16 | -2.43913 | 3.65E-06 |
| GALNT16 | 3.148004 | 3.66E-07 | 4.283643 | 1.58E-13 |  | **DGCR9** | -3.39904 | 0.016378 | -2.94545 | 0.021433 |
| GPR158 | 3.899114 | 8.55E-16 | 5.768237 | 1.44E-35 |  | **EPHX2** | -5.55057 | 8.33E-05 | -3.51782 | 0.000225 |
| GPR37 | 2.662682 | 9.25E-10 | 2.309835 | 2.70E-07 |  | **FAM107A** | -2.02718 | 0.002319 | -3.7692 | 2.07E-07 |
| GPR85 | 2.309996 | 4.18E-05 | 3.324937 | 2.04E-10 |  | **FAM83H** | -2.04926 | 0.009811 | -5.00905 | 4.94E-11 |
| GYPC | 4.089543 | 6.02E-08 | 4.842865 | 5.20E-11 |  | **FANK1** | -2.19536 | 2.60E-06 | -2.31613 | 3.76E-07 |
| HAS2 | 2.095249 | 0.000402 | 2.225 | 0.00019 |  | **FOLR1** | -2.50865 | 1.70E-05 | -4.52412 | 1.19E-13 |
| HOXB-AS3 | 5.299013 | 8.75E-07 | 5.241859 | 1.54E-06 |  | **FPR1** | -3.86645 | 1.50E-08 | -2.47377 | 0.000996 |
| HOXB5 | 3.452698 | 0.000427 | 3.711689 | 0.000129 |  | **FPR2** | -2.75816 | 0.003794 | -2.44323 | 0.007227 |
| HOXB6 | 4.200614 | 1.10E-06 | 4.212636 | 1.20E-06 |  | **GASK1B** | -4.78971 | 3.02E-10 | -3.436 | 1.88E-10 |
| IGFN1 | 3.622358 | 0.033476 | 5.960148 | 4.81E-05 |  | **GGT1** | -2.47566 | 0.026172 | -3.28449 | 0.004232 |
| IGSF1 | 6.508981 | 5.58E-06 | 8.922789 | 4.77E-11 |  | **GSN** | -2.42129 | 2.00E-06 | -2.0578 | 0.000124 |
| IGSF3 | 2.289026 | 1.63E-17 | 2.950581 | 2.55E-29 |  | **GYG2** | -2.35026 | 6.17E-08 | -2.68215 | 4.15E-10 |
| IGSF9B | 3.955691 | 0.000491 | 5.547218 | 1.13E-07 |  | **HSD3B1** | -9.15839 | 1.32E-11 | -9.49629 | 2.56E-12 |
| IL13RA2 | 9.14031 | 6.48E-11 | 10.30288 | 1.05E-13 |  | **HSPB8** | -2.7658 | 0.000212 | -3.74582 | 3.49E-07 |
| IL1B | 3.956212 | 2.64E-13 | 6.53043 | 5.90E-37 |  | **HTRA3** | -2.05094 | 0.002734 | -3.80581 | 2.57E-09 |
| IL6R | 3.656655 | 1.14E-10 | 4.370254 | 1.90E-15 |  | **IFI27** | -4.67237 | 3.35E-15 | -3.24919 | 7.66E-08 |
| IL7 | 3.014296 | 0.004501 | 2.636202 | 0.02362 |  | **IGFL2-AS1** | -3.82769 | 0.000177 | -4.82768 | 4.84E-05 |
| INSYN2B | 2.080155 | 2.52E-08 | 2.185547 | 5.07E-09 |  | **IGHV1-14** | -5.89837 | 0.002396 | -6.23626 | 0.001588 |
| IQGAP2 | 2.883014 | 7.61E-06 | 3.712 | 1.46E-09 |  | **IGHV3-15** | -7.17577 | 1.64E-06 | -7.51366 | 6.02E-07 |
| KAZN-AS1 | 5.146223 | 3.15E-15 | 4.041263 | 3.32E-09 |  | **IL1RN** | -3.21687 | 0.009685 | -4.51501 | 0.003367 |
| KBTBD8 | 5.576101 | 8.04E-14 | 2.879463 | 0.001098 |  | **IL32** | -2.70587 | 5.29E-05 | -2.90449 | 1.24E-05 |
| KCNJ12 | 4.948562 | 0.033552 | 5.042796 | 0.036963 |  | **IQCA1** | -3.94483 | 0.029964 | -6.05323 | 0.00093 |
| KCNQ3 | 3.230963 | 1.96E-08 | 3.736095 | 3.12E-11 |  | **JAG2** | -2.64787 | 0.002077 | -2.20575 | 0.016518 |
| KIF6 | 3.956969 | 0.001141 | 4.518268 | 0.000135 |  | **JCAD** | -2.81083 | 8.66E-33 | -3.7287 | 2.53E-52 |
| KISS1 | 4.015309 | 2.23E-10 | 4.151942 | 5.84E-11 |  | **KEL** | -5.18766 | 0.012826 | -5.52556 | 0.009274 |
| KRT17 | 2.722048 | 0.013488 | 3.310851 | 0.001588 |  | **KIAA1211L** | -6.25035 | 0.000112 | -3.30482 | 0.001643 |
| KRTAP2-3 | 3.125401 | 1.88E-09 | 2.938493 | 2.69E-08 |  | **KIF26B** | -3.48596 | 7.03E-12 | -2.00703 | 2.08E-06 |
| LAMA1 | 3.517763 | 1.78E-20 | 3.195441 | 8.20E-17 |  | **KIR3DX1** | -6.30784 | 5.47E-05 | -7.60753 | 6.79E-07 |
| LCT | 5.573949 | 0.001167 | 4.877488 | 0.00929 |  | **KNDC1** | -2.77375 | 0.003978 | -4.77549 | 0.000116 |
| LCTL | 2.845414 | 6.46E-09 | 3.418077 | 3.72E-13 |  | **LAIR2** | -7.65513 | 6.39E-08 | -6.14256 | 1.85E-06 |
| LGALS9C | 5.546998 | 2.19E-05 | 6.664966 | 1.59E-07 |  | **LBH** | -3.37378 | 5.47E-16 | -4.26365 | 2.07E-24 |
| LINC00355 | 2.030179 | 0.001533 | 2.419242 | 6.77E-05 |  | **LGALS12** | -8.68206 | 1.50E-27 | -11.2404 | 5.41E-17 |
| LINC00632 | 8.056528 | 2.49E-14 | 3.675366 | 0.004716 |  | **LINC00106** | -2.3508 | 0.005181 | -2.4455 | 0.003458 |
| LINC01111 | 2.666639 | 0.008431 | 2.35239 | 0.034525 |  | **LINC00525** | -4.19863 | 0.010137 | -2.87921 | 0.031807 |
| LINC01139 | 4.993089 | 7.18E-51 | 4.906139 | 3.57E-49 |  | **LINC00589** | -2.32831 | 0.001151 | -2.7891 | 8.69E-05 |
| LINC01291 | 4.069655 | 4.76E-07 | 2.828086 | 0.001833 |  | **LINC00639** | -2.7872 | 1.63E-05 | -2.58264 | 3.46E-05 |
| LINC01293 | 3.794321 | 6.16E-06 | 2.680908 | 0.004753 |  | **LINC01085** | -3.91281 | 3.22E-11 | -3.06269 | 7.15E-08 |
| LINC02241 | 6.529503 | 1.88E-05 | 5.502711 | 0.0008 |  | **LINC01088** | -2.79689 | 6.79E-05 | -2.88178 | 2.10E-05 |
| LINC02377 | 6.759105 | 7.55E-06 | 4.682233 | 0.008579 |  | **LINC01203** | -3.40343 | 1.76E-05 | -4.01978 | 1.34E-06 |
| LINC02389 | 4.863227 | 0.018655 | 5.004919 | 0.018104 |  | **LINC01770** | -5.24801 | 0.017115 | -5.5859 | 0.013032 |
| LMO2 | 4.101236 | 1.19E-19 | 3.344113 | 7.77E-13 |  | **LINC02009** | -2.2224 | 1.63E-08 | -4.28899 | 1.97E-28 |
| LNCOG | 2.997673 | 3.15E-15 | 2.636607 | 1.08E-11 |  | **LRRC61** | -2.57097 | 0.00134 | -3.68346 | 1.11E-06 |
| March4 | 2.521269 | 1.62E-19 | 2.933895 | 1.70E-26 |  | **LUM** | -7.28229 | 6.02E-07 | -3.61415 | 1.51E-05 |
| MIR137HG | 2.163711 | 2.23E-05 | 2.068512 | 8.25E-05 |  | **MUC1** | -5.5863 | 6.68E-26 | -2.96874 | 1.04E-10 |
| MIR4697HG | 2.247466 | 0.023327 | 4.454053 | 2.63E-08 |  | **MVB12B** | -3.96826 | 8.17E-22 | -2.30053 | 1.19E-08 |
| MMP1 | 7.734544 | 1.49E-124 | 6.367883 | 1.32E-83 |  | **MYCBPAP** | -5.38257 | 0.00073 | -2.10772 | 0.035243 |
| MMP3 | 7.175342 | 3.45E-07 | 7.060606 | 7.78E-07 |  | **NACA4P** | -2.44202 | 0.027545 | -2.25215 | 0.041171 |
| MNX1-AS1 | 3.010473 | 5.28E-05 | 2.558564 | 0.001377 |  | **NXF3** | -2.599 | 0.001155 | -2.7323 | 0.00048 |
| MPP4 | 4.098171 | 5.34E-57 | 4.149019 | 1.54E-58 |  | **NYAP2** | -2.75832 | 0.00134 | -5.45504 | 8.16E-05 |
| MSI2 | 3.459138 | 2.04E-24 | 3.256367 | 1.55E-21 |  | **PBX1** | -3.94798 | 2.78E-57 | -2.73695 | 1.21E-29 |
| MSX2 | 2.65172 | 4.64E-08 | 2.675933 | 3.85E-08 |  | **PCDH1** | -3.25842 | 8.60E-13 | -3.01406 | 2.23E-11 |
| MUC15 | 6.606798 | 3.77E-05 | 6.879551 | 1.99E-05 |  | **PI16** | -3.25525 | 0.000346 | -4.56474 | 1.32E-06 |
| MUC5B | 4.19171 | 4.82E-06 | 3.462756 | 0.000426 |  | **PLAAT2** | -2.83301 | 0.027438 | -3.32037 | 0.011026 |
| MYO1D | 2.308472 | 1.40E-08 | 3.712135 | 9.95E-23 |  | **PLAAT4** | -3.91307 | 2.01E-11 | -3.2347 | 6.96E-08 |
| MYO7B | 3.737159 | 4.35E-17 | 2.285235 | 2.87E-06 |  | **PLAAT5** | -6.41476 | 2.74E-07 | -8.58012 | 1.05E-09 |
| NCKAP1L | 4.962081 | 0.002577 | 5.449797 | 0.000842 |  | **PLEKHS1** | -2.32749 | 6.65E-14 | -4.93041 | 1.97E-37 |
| NELL2 | 2.308341 | 0.000505 | 3.816601 | 4.04E-11 |  | **PLXNA4** | -3.22086 | 5.72E-25 | -2.14366 | 1.07E-12 |
| NKX1-2 | 3.422465 | 0.006567 | 3.926147 | 0.001259 |  | **PRDM16** | -7.49406 | 3.18E-07 | -2.12185 | 0.008323 |
| NLRP10 | 2.214767 | 5.08E-05 | 2.231986 | 4.98E-05 |  | **PRELP** | -2.94679 | 0.000491 | -3.20729 | 0.000123 |
| NPL | 2.983707 | 3.60E-08 | 2.297016 | 8.22E-05 |  | **PRSS2** | -2.22343 | 0.006213 | -7.86784 | 1.03E-07 |
| NRG2 | 3.321348 | 3.60E-06 | 2.272907 | 0.00578 |  | **PSG1** | -6.83023 | 3.40E-08 | -5.83435 | 2.18E-11 |
| OR10A3 | 3.926398 | 0.005615 | 3.589244 | 0.018863 |  | **PSG5** | -2.99762 | 1.20E-11 | -3.19815 | 1.76E-13 |
| OR5E1P | 3.88319 | 0.004886 | 3.316265 | 0.031278 |  | **RAB7B** | -5.21312 | 0.000193 | -2.21177 | 0.025017 |
| OVCH2 | 4.163311 | 6.50E-09 | 3.829802 | 1.89E-07 |  | **RARRES2** | -6.88236 | 6.22E-32 | -5.52109 | 1.19E-26 |
| P3H3 | 3.510461 | 3.46E-17 | 2.494577 | 1.56E-08 |  | **RBM47** | -4.73745 | 4.22E-61 | -3.5221 | 4.79E-44 |
| PADI4 | 3.613615 | 0.01555 | 8.445792 | 1.87E-12 |  | **RFLNA** | -7.00099 | 3.72E-05 | -3.37478 | 0.009932 |
| PAPPA | 2.45028 | 1.12E-19 | 3.994656 | 3.30E-53 |  | **RNASE1** | -4.46137 | 2.58E-06 | -7.03668 | 2.54E-06 |
| PARM1 | 2.544528 | 2.00E-19 | 2.764054 | 2.79E-23 |  | **RNF224** | -4.27909 | 6.55E-05 | -2.79758 | 0.00136 |
| PCDHB2 | 3.942317 | 8.41E-08 | 3.200009 | 4.56E-05 |  | **RSAD2** | -4.99887 | 1.17E-08 | -4.01961 | 2.57E-09 |
| PCSK5 | 5.791882 | 2.10E-30 | 3.287729 | 2.75E-09 |  | **SCNN1A** | -5.03452 | 1.69E-39 | -3.2561 | 1.02E-18 |
| PDE1C | 4.696793 | 3.90E-34 | 2.843895 | 2.49E-12 |  | **SEMA5A** | -3.96621 | 1.81E-17 | -2.96118 | 6.55E-12 |
| PDGFD | 5.544649 | 2.03E-10 | 5.014468 | 2.02E-08 |  | **SERPINA1** | -6.32048 | 1.03E-36 | -2.48897 | 1.44E-07 |
| PHACTR1 | 4.088332 | 3.24E-33 | 2.782707 | 9.26E-15 |  | **SLC13A3** | -2.89616 | 8.43E-05 | -2.35307 | 0.00108 |
| PHGDH | 3.444309 | 7.85E-11 | 3.944423 | 5.31E-14 |  | **SLC29A4** | -2.22863 | 0.006085 | -2.35487 | 0.003652 |
| PIEZO2 | 4.187236 | 5.33E-06 | 2.397658 | 0.041171 |  | **SLC30A10** | -6.69956 | 2.03E-08 | -2.0613 | 4.22E-05 |
| PIK3CG | 4.192116 | 3.35E-10 | 4.982972 | 1.57E-14 |  | **SLC34A3** | -2.13051 | 0.015472 | -2.55806 | 0.003206 |
| PLCB1 | 6.350438 | 2.64E-20 | 6.753698 | 5.83E-23 |  | **SLC7A4** | -7.3354 | 1.44E-06 | -3.62446 | 0.000146 |
| PLCXD2 | 2.549851 | 2.73E-08 | 2.025319 | 3.10E-05 |  | **SMKR1** | -2.11407 | 0.020619 | -2.22743 | 0.01467 |
| PLXDC2 | 4.158252 | 2.57E-21 | 2.246941 | 4.55E-06 |  | **SNCG** | -3.38285 | 3.56E-13 | -2.1534 | 3.03E-06 |
| POU3F2 | 5.051124 | 3.22E-11 | 4.505836 | 7.91E-09 |  | **SORL1** | -4.11172 | 1.00E-65 | -3.56671 | 2.66E-54 |
| PPM1E | 2.496592 | 4.71E-11 | 2.627985 | 3.29E-12 |  | **SOST** | -3.94366 | 1.58E-05 | -3.92458 | 1.27E-05 |
| PRRX1 | 2.540166 | 4.26E-05 | 3.371413 | 8.02E-09 |  | **SPINK4** | -2.34007 | 5.41E-05 | -6.47437 | 1.03E-16 |
| PTK7 | 2.988084 | 1.88E-09 | 2.211108 | 3.27E-05 |  | **SPON2** | -2.51828 | 0.011846 | -3.03946 | 0.002247 |
| PTPRN2 | 5.018439 | 2.31E-33 | 6.12104 | 3.10E-50 |  | **SUSD2** | -2.00719 | 0.000527 | -4.28343 | 4.13E-10 |
| PURPL | 6.062476 | 3.34E-07 | 4.259866 | 0.001404 |  | **SYT12** | -3.38598 | 1.88E-07 | -2.0978 | 0.002639 |
| RAPGEF5 | 3.141827 | 2.28E-06 | 2.953553 | 1.46E-05 |  | **TBC1D29P** | -3.58327 | 0.000168 | -2.7196 | 0.002119 |
| RASEF | 5.264544 | 3.55E-13 | 4.267318 | 1.50E-08 |  | **TCN1** | -4.36732 | 9.34E-07 | -2.50884 | 9.34E-05 |
| RERG | 2.785289 | 0.006837 | 2.407256 | 0.035071 |  | **TLR8-AS1** | -2.27411 | 3.88E-06 | -4.65525 | 6.61E-12 |
| RNF150 | 2.025053 | 0.006305 | 2.663073 | 0.000101 |  | **TMEM130** | -6.59798 | 2.88E-05 | -4.10327 | 0.000829 |
| RNU1-59P | 5.234424 | 8.82E-07 | 3.977702 | 0.000695 |  | **TNFSF10** | -4.20233 | 5.15E-41 | -4.19536 | 2.24E-42 |
| ROBO1 | 3.12538 | 2.97E-14 | 2.633898 | 4.59E-10 |  | **TNS4** | -3.94281 | 1.04E-19 | -2.91447 | 3.50E-11 |
| ROBO2 | 8.977496 | 1.82E-10 | 4.221962 | 0.019144 |  | **TP53I11** | -3.47542 | 9.72E-11 | -12.0914 | 5.80E-19 |
| RTN1 | 2.642917 | 3.84E-08 | 4.371521 | 2.57E-23 |  | **TRBC1** | -5.80779 | 0.001566 | -4.26905 | 0.024174 |
| SAMD5 | 3.080241 | 9.33E-07 | 2.47386 | 0.000233 |  | **TRBC2** | -6.42955 | 4.15E-05 | -7.72923 | 4.90E-07 |
| SEL1L3 | 2.343353 | 5.91E-11 | 2.272722 | 2.94E-10 |  | **TRBV6-5** | -4.54143 | 0.045657 | -5.84112 | 0.006837 |
| SERPINB2 | 5.291092 | 4.53E-13 | 2.415124 | 0.008899 |  | **TRIM29** | -3.24198 | 1.39E-08 | -7.38566 | 9.14E-13 |
| SETBP1 | 5.224936 | 3.81E-11 | 4.070254 | 1.22E-06 |  | **TSPOAP1** | -2.0691 | 3.21E-09 | -3.61331 | 4.80E-25 |
| SH2D2A | 4.047295 | 1.20E-07 | 3.431558 | 1.95E-05 |  | **TTLL2** | -3.63763 | 0.01121 | -3.89313 | 0.00657 |
| SIDT1 | 2.794693 | 4.85E-05 | 2.302425 | 0.0021 |  | **UCA1** | -2.85208 | 3.87E-09 | -5.53111 | 2.80E-20 |
| SLC19A3 | 3.100536 | 1.47E-05 | 3.258855 | 4.44E-06 |  | **UNC5B** | -2.50918 | 0.007986 | -2.09297 | 0.033661 |
| SLC22A18AS | 2.015104 | 0.001389 | 3.440076 | 9.46E-10 |  | **VSTM4** | -8.4459 | 0.001131 | -5.71776 | 0.031332 |
| SMOC1 | 4.66392 | 0.013363 | 4.689838 | 0.015838 |  | **VTN** | -2.25471 | 0.021846 | -2.30262 | 0.019385 |
| SNCAIP | 4.001148 | 2.03E-15 | 4.249215 | 1.97E-17 |  | **VWA1** | -2.08167 | 0.033005 | -2.94544 | 0.001162 |
| SOBP | 5.25129 | 1.52E-11 | 5.870799 | 2.10E-14 |  | **ZBED6CL** | -3.30082 | 4.82E-13 | -4.4217 | 7.68E-23 |
| SPARC | 3.253893 | 2.29E-11 | 7.865224 | 1.39E-66 |  | **ZP3** | -6.48511 | 1.35E-05 | -2.64479 | 0.000486 |
| SPOCK1 | 2.542991 | 4.11E-20 | 3.018428 | 9.26E-29 |  |  |  |  |  |  |
| SRGN | 3.03207 | 8.74E-45 | 3.364145 | 4.76E-55 |  |  |  |  |  |  |
| SYPL2 | 2.279948 | 1.92E-08 | 2.057542 | 7.86E-07 |  |  |  |  |  |  |
| SYT16 | 2.536013 | 0.011899 | 3.091074 | 0.001481 |  |  |  |  |  |  |
| SYT9 | 6.952216 | 9.89E-06 | 4.2042 | 0.036503 |  |  |  |  |  |  |
| TENM1 | 6.414775 | 2.93E-21 | 5.919295 | 5.68E-18 |  |  |  |  |  |  |
| TENM2 | 8.100362 | 2.43E-16 | 4.065069 | 0.000426 |  |  |  |  |  |  |
| TENM4 | 9.200128 | 1.23E-11 | 5.022467 | 0.002008 |  |  |  |  |  |  |
| TEX41 | 5.334664 | 5.10E-05 | 5.878226 | 6.45E-06 |  |  |  |  |  |  |
| TFAP2C | 2.128245 | 3.17E-18 | 2.82803 | 2.29E-32 |  |  |  |  |  |  |
| TIE1 | 7.622397 | 8.74E-08 | 5.244875 | 0.001067 |  |  |  |  |  |  |
| TLE2 | 3.053881 | 3.46E-05 | 2.620959 | 0.000882 |  |  |  |  |  |  |
| TMEM40 | 2.612503 | 0.000255 | 4.520674 | 7.77E-13 |  |  |  |  |  |  |
| TRIML2 | 2.216306 | 2.45E-16 | 2.461659 | 1.93E-20 |  |  |  |  |  |  |
| TTPA | 3.053839 | 0.000523 | 2.843609 | 0.00207 |  |  |  |  |  |  |
| WDR72 | 4.188069 | 3.57E-17 | 5.092218 | 1.96E-25 |  |  |  |  |  |  |
| WNT5A | 5.498562 | 6.26E-15 | 3.112453 | 0.000104 |  |  |  |  |  |  |
| WTAPP1 | 4.863381 | 0.012922 | 4.410824 | 0.039871 |  |  |  |  |  |  |
| ZG16B | 3.614537 | 9.48E-10 | 2.194479 | 0.001127 |  |  |  |  |  |  |
| ZNF804A | 2.906171 | 3.23E-05 | 2.02163 | 0.011889 |  |  |  |  |  |  |
