# Supplementary material for: Comparative analysis of protein-protein interaction networks in metastatic breast cancer
Source: PLoS One. 2022 Jan 19;17(1):e0260584. doi: 10.1371/journal.pone.0260584 (PMC8769308; doi:10.1371/journal.pone.0260584)
Supplement: S6 Table — Which are represented (A) Clusters obtained from brain metastatic breast cancer PPI network. (B) Clusters obtained from lung metastatic breast cancer PPI network. (DOCX) [file pone.0260584.s008.docx]

**S6. Table. Ranked clusters of PPI networks analyzed by MCODE**

| A: PPI protein complex associated with brain metastatic breast cancer | | | | |
| --- | --- | --- | --- | --- |
| Cluster | Score | Nodes | Edges | Gene symbols |
| 1 | 19 | 19 | 171 | S1PR5, S1PR3, HTR1F, GRM8, GPR55, GPR37, GNGT2, GNG2, GABBR2, FPR2, FPR1, DRD2, CXCR4, CXCL8, CXCL16, CX3CL1, C3, ADRA2C, ACKR3 |
| 2 | 9.333 | 10 | 42 | PLCB4, PLCB2, PLCB1, KISS1, HTR2C, GRPR, F2RL3, F2RL2, CHRM3, ARHGEF25 |
| 3 | 9 | 9 | 36 | THBS2, SPON2, SEMA5A, CFP, ADAMTSL3, ADAMTSL2, ADAMTS5, ADAMTS14, ADAMTS1 |
| 4 | 9 | 9 | 36 | TRIM9, SOCS1, RNF144B, KLHL3, KLHL13, KBTBD8, FBXL7, FBXL16, ASB2 |
| 5 | 9 | 9 | 36 | WNT5A, SYT9, SYT8, SYT11, STON2, LRP2, EGF, AVPR2, AMPH |
| 6 | 8 | 8 | 28 | VNN1, THY1, TECTA, MDGA1, LYPD3, LY6K, LSAMP, CNTN5 |
| 7 | 7 | 15 | 49 | ST6GALNAC3, ST6GALNAC2, ST6GAL1, MUC5B, MUC4, MUC15, MUC1, LUM, GALNT16, GALNT15, GALNT14, GALNT12, B3GNT8, B3GNT7, B3GNT3 |
| 8 | 5.417 | 25 | 67 | VWA1, TCN1, SRGN, SPARC, SERPING1, SERPINA1, PTPN6, P4HA3, MMP3, MMP1, METTL7A, LYZ, LEPREL2, IL1B, IGFBP1, CTSD, COL8A2, COL7A1, COL5A1, COL1A2, COL18A1, COL11A1, CLU, CHRDL1, CCL2 |
| B: PPI protein complex associated with lung metastatic breast cancer | | | | |
| Cluster | Score | Nodes | Edges | Gene symbols |
| 1 | 15 | 15 | 105 | SAA1, HTR1D, HTR1B, GPR37, GABBR2, FPR3, FPR2, FPR1, CXCR4, CXCL16, CXCL11, CX3CL1, CCR5, CCR3, CCL16 |
| 2 | 8 | 8 | 28 | PTGFR, PLCB1, P2RY6, P2RY2, LPAR6, KISS1, FFAR4, CHRM3 |
| 3 | 5 | 5 | 10 | VWA1, SERPINA1, MXRA8, MFI2, AFP |
| 4 | 5 | 5 | 10 | SPON2, SEMA5A, CFP, ADAMTS17, ADAMTS15 |

Which are represented (A) Clusters obtained from brain metastatic breast cancer PPI network. (B) Clusters obtained from lung metastatic breast cancer PPI network.
