# Supplementary material for: Comparative analysis of protein-protein interaction networks in metastatic breast cancer
Source: PLoS One. 2022 Jan 19;17(1):e0260584. doi: 10.1371/journal.pone.0260584 (PMC8769308; doi:10.1371/journal.pone.0260584)
Supplement: S9 Table — Which are implemented on the identified hub genes in the MDA-MB-231 cell line. (*) indicates the genes that are shared between Brain and Lung tumour metastases. (DOCX) [file pone.0260584.s011.docx]

**S9. Table.** **CRISPR and RNAi represent the gene knockout methods.**

Which are implemented on the identified hub genes in MDA-MB-231 cell line.

(*) indicates the genes that are shared between Brain and Lung tumor metastases.

| **A. Hub genes in the brain metastatic breast cancer** | | | | **B. Hub genes in the Lung metastatic breast cancer** | | | |
| --- | --- | --- | --- | --- | --- | --- | --- |
| **Genes** | **Expression log2(TPM+1)** | **CRISPER** | **RNAi** | **Genes** | **Expression log2(TPM+1)** | **CRISPER** | **RNAi** |
| **GNG2** | 0.594549 | -0.40136 | 0.10517 | **FPR2*** | 0.516015 | 0.135885 | -0.17205 |
| **GNGT2** | 0.505891 | 0.120232 | -0.01611 | **CXCR4*** | 3.054848 | 0.045999 | 0.332823 |
| **CXCR4*** | 3.054848 | 0.045999 | 0.332823 | **SAA1** | 5.751678 | -0.15699 | 0.083486 |
| **FPR2*** | 0.516015 | 0.135885 | -0.17205 | **CCR5** | 0.028569 | -0.25776 | 0.06239 |
| **C3** | 1.389567 | -0.30955 | -0.08733 | **FPR1*** | 3.238787 | 0.209295 | 0.032103 |
| **EGF** | 1.22033 | 0.170635 | 0.169188 | **CCR3** | 0 | -0.21192 | 0.092107 |
| **CXCL8** | 2.124328 | -0.02635 | -0.02922 | **CX3CL1*** | 1.646163 | 0.282768 | -0.16657 |
| **MMP9** | 0.028569 | -0.08911 | -0.02332 | **CXCL11** | 1.104337 | 0.019007 | -0.04617 |
| **CX3CL1*** | 1.646163 | 0.282768 | -0.16657 | **GPR37*** | 0.014355 | 0.284632 | 0.003017 |
| **FPR1*** | 3.238787 | 0.209295 | 0.032103 | **GABBR2*** | 0.028569 | -0.19433 | -0.13975 |
| **CXCL16** | 1.906891 | -0.08351 | -0.39595 | **PLCB1*** | 0.214125 | 0.253985 | 0.089316 |
| **GPR55** | 0.250962 | 0.052317 | -0.13672 |  |  |  |  |
| **WNT5A** | 0.084064 | -0.23931 | -0.10412 |  |  |  |  |
| **GABBR2*** | 0.028569 | -0.19433 | -0.13975 |  |  |  |  |
| **S1PR5** | 0.613532 | -0.02612 | -0.01357 |  |  |  |  |
| **ADRA2C** | 0.516015 | 0.112298 | -0.13783 |  |  |  |  |
| **DRD2** | 0.495695 | 0.05348 | NA |  |  |  |  |
| **TIMP1** | 8.990671 | 0 | 0.004351 |  |  |  |  |
| **GPR37*** | 0.014355 | 0.284632 | 0.003017 |  |  |  |  |
| **S1PR3** | 1.339137 | 0.004919 | 0.152616 |  |  |  |  |
| **ACKR3** | 1.937344 | 0.139391 | -0.19615 |  |  |  |  |
| **PLCB2** | 0.739848 | 0.105394 | -0.20033 |  |  |  |  |
| **AVPR2** | 0.056584 | 0 | 0.035029 |  |  |  |  |
| **PLCB4** | 0.042644 | -0.10305 | 0.107017 |  |  |  |  |
| **GRM8** | 0.028569 | 0.008932 | -0.17151 |  |  |  |  |
| **PLCB1*** | 0.214125 | 0.253985 | 0.089316 |  |  |  |  |
| **HTR1F** | 0 | -0.2688 | 0.023424 |  |  |  |  |
| **COL18A1** | 5.940167 | 0.0622 | 0.003822 |  |  |  |  |
| **PTPN6** | 2.620586 | 0.100916 | 0.110955 |  |  |  |  |
| **GNAO1** | 2.277985 | -0.15263 | -0.07263 |  |  |  |  |
| **ITGB2** | 2.244887 | 0.102243 | -0.03184 |  |  |  |  |
| **HTR2C** | 0.014355 | 0 | 0.013752 |  |  |  |  |
| **CFP** | 3.729009 | 0 | 0.156751 |  |  |  |  |
| **LRP2** | 0.344829 | -0.10216 | 0.058981 |  |  |  |  |
