# Supplementary material for: Comparative analysis of protein-protein interaction networks in metastatic breast cancer
Source: PLoS One. 2022 Jan 19;17(1):e0260584. doi: 10.1371/journal.pone.0260584 (PMC8769308; doi:10.1371/journal.pone.0260584)
Supplement: S10 Table — The Type column represents where genes are differentially expressed as specific which is expressed in the brain and doesn’t express in Lung and which are differentially expressed in both Lung and Brain tumours that metastasis from Breast cancer. The Name column showed the Gene Symbol of each gene and HR the column represents Hazard Ratio value and also log-rank P column showed each significant (log-rank P = < 0.05) values. (DOCX) [file pone.0260584.s012.docx]

**S10 Table. Survival analysis to three types of Hub genes.** The Type column represent where genes differentially expressed as specific which is expressed in the brain and doesn’t express in Lung and which are differentially expressed in both Lung and Brain tumours that metastasis from Breast cancer. The Name column showed the Gene Symbol of each gene and HR the column represents Hazard Ratio value and also log-rank P column showed each significant (log-rank P =< 0.05) values.

| Type | Name | HR | logrank P |
| --- | --- | --- | --- |
| Brain | GNG2 | 0.56 | 3.50E-07 |
|  | CXCL8 | 1.47 | 6.60E-05 |
|  | PTPN6 | 0.7 | 2.30E-04 |
|  | C3 | 0.67 | 3.20E-05 |
|  | PLCB2 | 0.79 | 0.012 |
|  | LRP2 | 0.66 | 2.80E-03 |
|  | ADRA2C | 1.28 | 1.10E-02 |
| Common | GPR37 | 1.31 | 0.0044 |
|  | FPR1 | 0.77 | 0.0074 |
| Lung | SAA1 | 0.8 | 0.017 |
|  | CCR5 | 0.68 | 4.90E-05 |
